# Opposite Effects Induced by Cholinium-Based Ionic Liquid Electrolytes in the Formation of Aqueous Biphasic Systems Comprising Polyethylene Glycol and Sodium Polyacrylate

**DOI:** 10.3390/molecules26216612

**Published:** 2021-10-31

**Authors:** Sandra C. Bernardo, Emanuel V. Capela, Jorge F. B. Pereira, Sónia P. M. Ventura, Mara G. Freire, João A. P. Coutinho

**Affiliations:** 1 CICS-UBI–Health Sciences Research Centre, University of Beira Interior, 6200-506 Covilhã, Portugal; sandracsbernardo@gmail.com; 2 CICECO–Aveiro Institute of Materials, Department of Chemistry, University of Aveiro, 3810-193 Aveiro, Portugal; emanuelcapela@ua.pt (E.V.C.); spventura@ua.pt (S.P.M.V.); 3 Univ Coimbra, CIEPQPF, Department of Chemical Engineering, Rua Sílvio Lima, Pólo II–Pinhal de Marrocos, 3030-790 Coimbra, Portugal; jfbpereira@eq.uc.pt

**Keywords:** liquid-liquid equilibrium, cholinium-based ionic liquids, polymeric aqueous biphasic systems, electrolytes, demixing behavior

## Abstract

Cholinium-based ionic liquids ([Ch]-based ILs) were investigated as electrolytes in the formation of aqueous biphasic systems (ABS) composed of polyethylene glycol (PEG) and sodium polyacrylate (NaPA) polymers. Both enhancement and decrease in the liquid-liquid demixing ability induced by electrolytes in PEG-NaPA aqueous biphasic systems were observed. It is shown that the ILs that most extensively partition to the PEG-rich phase tend to act as inorganic salts enhancing the two-phase formation ability, while those that display a more significant partition to the NaPA-rich phase decrease the ABS formation capacity. The gathered results allowed us to confirm the tailoring ability of ILs and to identify, for the first time, opposite effects induced by electrolytes on the PEG-NaPA ABS formation ability. The distribution of the electrolyte ions between the coexisting phases and the polyelectrolyte ion compartmentalization are key factors behind the formation of PEG-NaPA-based ABS.

## 1. Introduction

Liquid-liquid extractions by means of aqueous biphasic systems (ABS) have been extensively explored in the last decades to recover, purify and/or concentrate a large number of biocompounds [1,2,3]. ABS are formed by the combination in water of at least two water-soluble compounds, (polymers, salts, ionic liquids, carbohydrates, among others) which act as phase-forming components [4,5]. Among these, polymer-polymer ABS have been the most studied since they have a high water content, and thus display a more biocompatible character, despite being restricted to only a few combinations of polymers [6,7,8,9,10,11].

A large number of water-soluble non-ionic polymers have been investigated as phase-forming agents of ABS; however, some of these polymers’ combinations do not undergo liquid-liquid demixing in aqueous media at convenient concentrations for separation purposes. The most commonly used polymer/-polymer pair for ABS formation is polyethylene glycol (PEG)-dextran [9,12], but dextran is more expensive in comparison with the polymers herein used and leads to highly viscous aqueous solutions, imposing some limitations of operability when the large-scale application of ABS is envisaged [13,14,15]. To overcome these shortcomings, polyelectrolytes have been investigated as phase-forming agents of ABS [16,17,18]. Due to the entropically driven dissociation of their counterions in aqueous solutions and consequent large contribution of the entropy of mixing, these charged polymers are highly water soluble [6]. In addition, polyelectrolyte aqueous solutions usually exhibit lower viscosity than non-ionic polymer solutions [10,18].

Although PEG and the polyelectrolyte sodium polyacrylate (NaPA) with adequate molecular weights can form ABS, they may not be able to create liquid-liquid systems at suitable polymer concentrations [6]. In order to overcome this drawback, the addition of inorganic salts (1–5 wt%) as electrolytes in PEG-NaPA ABS is commonly used [16,18,19]. The addition of salts decreases the entropic penalty of the polyelectrolyte counterions compartmentalization, favouring phase separation [6]. The decreased polymer content, while increasing the water amount, further decreases the overall system cost and the phases’ viscosities [11,18,19].

The effect of salts/electrolytes in the PEG-NaPA-water phase diagrams has been investigated by several authors, with some of these works focusing on a more fundamental perspective aiming to better understand the molecular-level phenomena ruling the phase separation. Gupta et al. [18] correlated the capability of ions as water structure-breakers or water structure-makers (kosmotropic vs. chaotropic salt ions) and their ability to induce ABS. Perrau et al. [11] and Johansson et al. [19] proposed that the salt addition decreases the entropy penalty of the polyelectrolyte counterion compartmentalization by charge effects. All these studies employed high-melting inorganic salts as additives/electrolytes, but when the application of these systems is foreseen for labile biomolecule separation, inorganic salts may raise some biocompatibility concerns [20,21].

Considering the relevance of ionic additives in the formation of polymer-polyelectrolyte ABS, ionic liquids (ILs) have been investigated as potential alternatives to the widely studied high-melting inorganic salts [22,23]. The use of ILs as additives, due to their tuneable characteristics, was shown to effectively control the formation of PEG-NaPA ABS. By adding tensioactive [23] and non-tensioactive ILs [22], it was possible to design ABS with different phase polarities and separation ability. However, the ILs used as electrolytes in previous works [22,23] may still raise some environmental and biocompatibility issues [24,25,26].

Aiming at developing liquid-liquid systems with enhanced environmentally friendly nature and biocompatible characteristics, in this work we investigated PEG-NaPA-based ABS using cholinium-based ILs ([Ch]-based ILs) as electrolytes. The effects of the PEG molecular weight (MW), electrolyte type and concentration on the formation ability of ABS were investigated at 300.15 K.

## 2. Results and Discussion

### 2.1. Effect of PEG Molecular Weight (MW)

**Figure 1:**
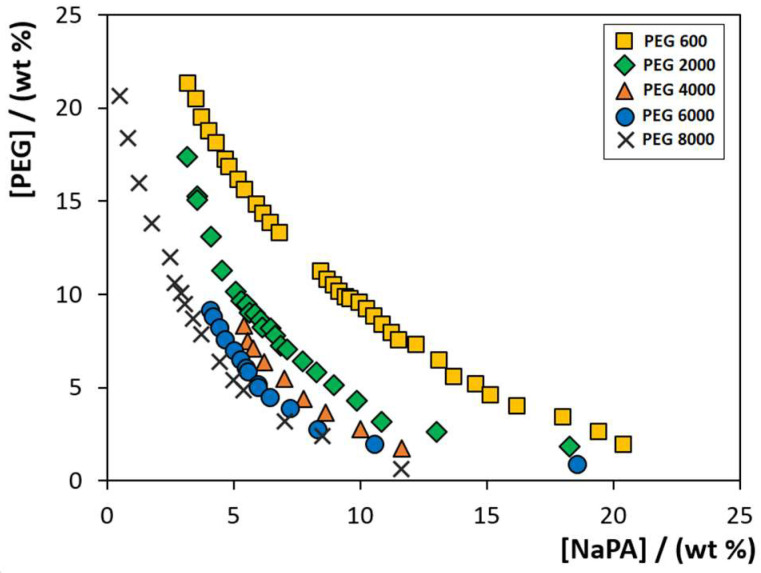
Solubility/binodal curves for ABS composed of PEG (different MW), NaPA 8000 + 5 wt% of [Ch]Cl, at 300.15 K: (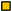) PEG 600; (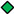) PEG 2000; (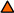) PEG 4000; (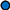) PEG 6000; and (**✕**) PEG 8000. Data corresponding to the latter system were taken from the literature [22].

To evaluate the PEG MW influence on the formation of PEG-NaPA-IL ABS, the phase diagrams composed of PEG polymers with different MW (600, 2000, 4000, 6000 and 8000 g·mol^−1^), NaPA with a MW of 8000 g·mol^−1^ and 5 wt% of each IL (cholinium chloride, [Ch]Cl; cholinium acetate, [Ch][Ac]; cholinium dihydrogenphosphate, [Ch][DHP]; cholinium dihydrogencitrate, [Ch][DHcit]; and cholinium bitartrate, [Ch][Bit]) were determined at 300.15 K by the cloud point titration method [27]. Figure 1 depicts the phase diagrams of the systems composed of PEG-NaPA + 5 wt% of [Ch]Cl. The remaining phase diagrams for systems containing the different [Ch]-based ILs combined with PEGs of different MW are given in Figure S1 in the Supplementary Material. The detailed weight fraction experimental data are provided in Tables S1–S5. Compositions of PEG and NaPA above each solubility curve, while keeping the concentration of ILs at 5 wt%, lead to the creation of two-phase systems, whereas compositions below the respective binodal curve result in a homogenous solution. Figure 1 shows an increase in the biphasic region, i.e., the ability to undergo liquid–liquid demixing with the PEG MW increase, and this phenomenon independent of the cholinium-based IL is used as an electrolyte. The same behaviour is obtained with other ILs or when representing the respective phase diagrams in molality units (see Figures S1 and S2 in the Supplementary Material).

In summary, PEGs with lower MW reduce the PEG-NaPA ABS biphasic region. Our results using [Ch]-based ILs closely agree with those previously reported using inorganic salts as electrolytes [8,19,28]. For all PEG-NaPA systems investigated, the biphasic region increases with the MW of PEG, meaning that lower amounts of phase-forming components are required to undergo liquid-liquid demixing when employing higher MW PEG polymers. The same trend has been reported for other ABS composed of PEG-polymer, [29] PEG-salt [30,31] and PEG-IL [32,33,34].

### 2.2. Effect of [Ch]−Based ILs Concentration in ABS Formation

**Figure 2:**
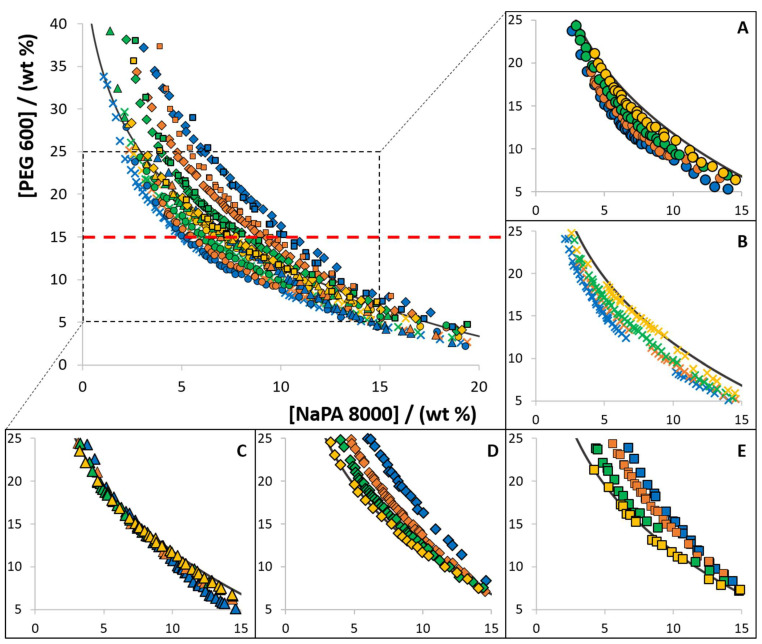
Experimental solubility data, at 300.15 K, for ABS composed of PEG 600, NaPA 8000, and each [Ch]-based IL used as an electrolyte: (**A**) [Ch][Ac] (**●**); (**B**) [Ch]Cl (**✕**); (**C**) [Ch][DHP] (**▲**); (**D**) [Ch][DHcit] (♦); and (**E**) [Ch][Bit] (■). Distinct colors represent different concentrations, namely 10 wt% (**blue**), 5 wt% (**orange**), 2.5 wt% (**green**), 1 wt% (**yellow**). The ABS formed by PEG 600 and NaPA 8000 without electrolyte is represented by the black line (**―**).

The influence of different [Ch]-based ILs as electrolytes in the phase diagram behaviour was investigated in the ABS composed of PEG 600, NaPA 8000 and water at 300.15 K. The ILs [Ch]Cl, [Ch][Ac], [Ch][DHP], [Ch][DHcit] and [Ch][Bit] were used as electrolytes at concentrations ranging from 0 to 10 wt%. The respective phase diagrams with PEG 600 are depicted in Figure 2. The detailed experimental weight fraction compositions are given in Tables S6–S14 in the Supplementary Material.

From the data shown in Figure 2, three different effects are identified when [Ch]-based ILs are used as electrolytes in ABS composed of NaPA 8000 and PEG 600: (i) increase in the biphasic region when increasing the concentration of [Ch]Cl and [Ch][Ac], acting as PEG-NaPA ABS formation enhancers; (ii) decrease in the biphasic region with the [Ch][DHcit] and [Ch][Bit] concentration increase, acting as ABS formation depressants; and (iii) no significant influence on the solubility/binodal curve of the PEG-NaPA ABS with [Ch][DHP]. It should be remarked that with [Ch][DHP], a slight inversion in the trend behaviour seems to occur according to the polymer concentration (changing from a type (i) to a type (ii) effect as the system moves from the NaPA-rich to the PEG-rich region).

All previous reports suggest that the addition of electrolytes leads to an increase in the two-phase region of PEG-NaPA ABS [8,19,28], as also confirmed in this work using NaCl and Na[Ac] (sodium chloride and sodium acetate, respectively) as electrolytes (data provided in the Supplementary Material—Figures S4 and S5). These differences in the PEG-NaPA ABS phase behaviour according to the electrolyte nature are shown here for the first time, supporting the IL tailoring ability and their more complex nature in terms of chemical nature and possibility of interactions when compared to high melting temperature salts [35,36].

The changes in the ABS formation ability are also dependent on the polymer concentration, with the electrolyte-inducing changes in the binodal curves being more evident at the PEG-rich region. Thus, it seems that a saturation effect of the added electrolyte occurs at the PEG-rich phase. As previously reported for inorganic salts [18,19], the IL (electrolyte) concentration used to promote the ABS formation is a key parameter.

### 2.3. Opposite Effects Explained: Determination of Electrolyte Partition

To fully understand the opposite effects promoted by [Ch]-based ILs, it is important to look into the mechanisms behind the formation of non-ionic + ionic polymer-based ABS. When using combinations of non-ionic and ionic polymers to create ABS, still without considering the addition of an electrolyte, a phase containing a high concentration of the polyelectrolyte and the corresponding counterions, and another enriched in the uncharged polymer will be created, with both being aqueous. In the absence of electrolytes or additives, the influence of the entropy of mixing of the polyelectrolyte counterions dominates the phases’ separation [6]. However, when an electrolyte is introduced, the molecular-level mechanisms which rule the phase equilibria are modified, and “the entropy of mixing of the counterions no longer dominates over the entropy of the polyions”, as discussed by Piculell and Lindman [6]. Previous works [11,18,19] provided two different explanations for the influence of electrolytes on the PEG-NaPA phase equilibria, but all reported positive deviations or the enlargement of the biphasic region by the addition of salts. However, based on the opposite trends observed herein with the ILs evaluated as electrolytes in the PEG 600-NaPA 8000 ABS, additional interpretations on the molecular-level mechanisms ruling the two-phase formation of systems composed of non-ionic and ionic polymers are required. To better address these phenomena, the distribution of the IL between the coexisting phases was experimentally determined.

**Figure 3:**
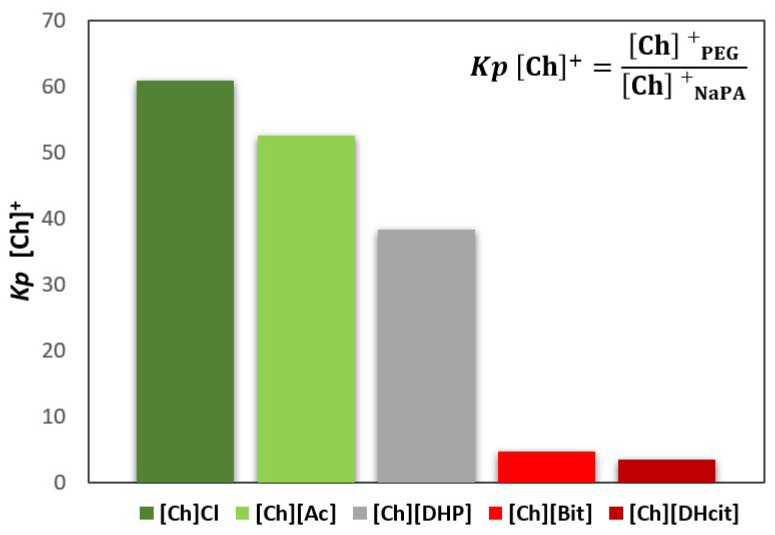
Cholinium cation partition coefficients (*Kp* [Ch]^+^), between the PEG-rich (**top**) phase and NaPA-rich (**bottom**) phase, determined for the systems, whereas [Ch]-based ILs were used as electrolytes in the formation of PEG 600 + NaPA 8000 + water + 10 wt% of [Ch]-ILs ABSs.

**Figure 4:**
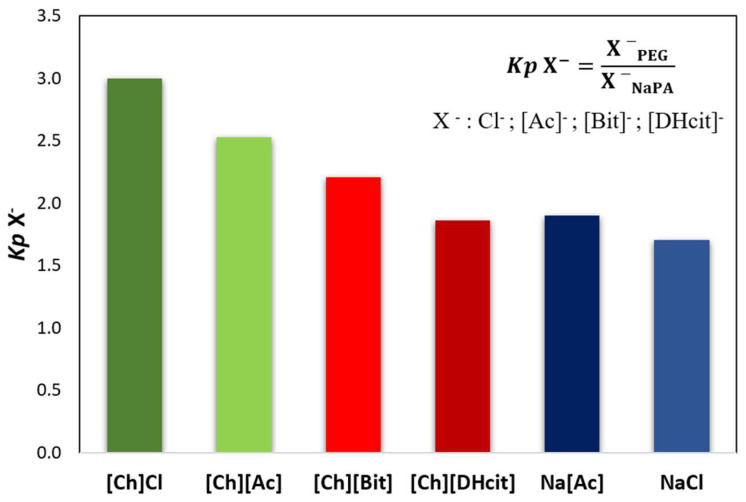
Anion partition coefficients (*Kp* X^−^), between the PEG-rich (**top**) phase and NaPA-rich (**bottom**) phase determined for the systems composed of PEG 600 + NaPA 8000 + water + 10 wt% of electrolytes, whereas X^−^ corresponds to chloride (Cl^−^), acetate ([Ac]^−^), bitartrate ([Bit]^−^) and dihydrogencitrate ([DHcit]^−^) anions of the added electrolytes.

For all the [Ch]-based ABS, a mixture point within the biphasic region was selected and the ABS prepared (3 g of total weight; 25 wt% of PEG 600 + 7.5 wt% of NaPA 8000 + 10 wt% of the [Ch]-ILs + 57.5% water). After equilibrium, the phases were separated, and the partition of the IL was determined by proton nuclear magnetic resonance (^1^H NMR; using C_6_H_6_ as internal standard). The [Ch]^+^ partition coefficients (*Kp* [Ch]^+^), defined as the ratio of the concentration of [Ch]^+^ in the PEG-rich phase to that in the NaPA-rich phase, are depicted in Figure 3 (additional experimental data are provided in the Supplementary Material—Table S23). The partition coefficients of some anions ([Ac]^−^, [Bit]^−^ and [DHcit]^−^) were determined by comparing the ^1^H NMR spectra of the two phases, while the partition coefficients of Cl^−^ were calculated by the ratio of the concentrations of Cl^−^ in the PEG-rich phase to that in the NaPA-rich phase, determined using a chloride ion-selective electrode. The partition coefficients of these anions (*Kp* X^−^) are depicted in Figure 4 (additional data are supplied in the Supplementary Material—Table S24).

Figure 3 and Figure 4 show that the IL ions partition towards the PEG-rich phase follows the trend: [Ch]Cl and [Ch][Ac] (*Kp* [Ch]^+^ > 50 and *Kp* X^−^ ≈ 3) >> [Ch][DHP] (*Kp* [Ch]^+^ ≈ 38) > [Ch][DHcit] and [Ch][Bit] (*Kp* [Ch]^+^ ≈ 4 and *Kp* X^−^ ≈ 2).

Although all the [Ch]-based ILs “prefer” more hydrophilic phases (octanol-water partition coefficients lower than 1 [33]), they have significantly different solubilities in water. Pereira et al. [33] showed that the solubility of [Ch]-based ILs in water (mol·kg^−1^) at 298.15 K follows the trend: [Ch]Cl (21.1) > [Ch][Ac] (20.9) > [Ch][DHP] (13.5) > [Ch][Bic] (4.8) > [Ch][DHcit] (3.2) [33]. Based on these results, the most water soluble ILs (i.e., with a more salting-in nature) are those that partition more extensively to the top(PEG)-rich phase. This trend agrees with literature data on the partition of inorganic salts in PEG-NaPA ABS, [19,34,35], in which more hydrophilic anions in terms of solvation free energy more extensively partition to the PEG-rich phase, as for example SO_4_^2−^ with a *K* elect = 1.0 vs. Cl^−^ with a *K* elect = 1.14 [19], as well as the previous data on the salting effect of different ammonium salts in aqueous polymer solutions [36]. Overall, it could be said that electrolytes that extensively partition to the PEG-rich phase act as ABS enhancers, whereas those that are less able to partition to the PEG-rich phase act as ABS depressants.

**Figure 5:**
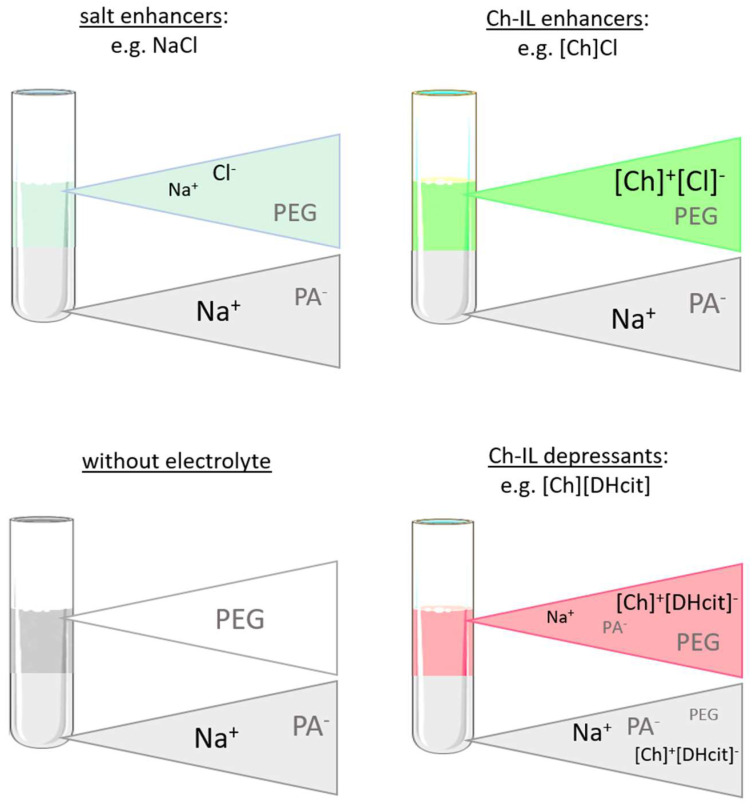
Schematic representation of partition coefficients of phase-forming components determined for the systems composed of PEG 600 + NaPA 8000 + water + 10 wt% of electrolyte and without electrolyte as a control, between the PEG-rich (**top**) phase and NaPA-rich (**bottom**) phase.

**Figure 6:**
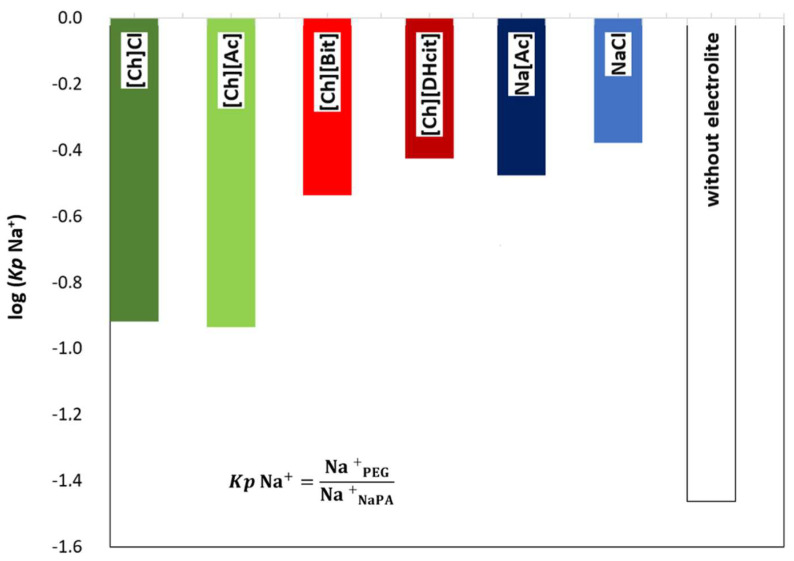
Logarithmic representation of sodium cation partition coefficients [log (*Kp* Na^+^)] between the PEG-rich (**top**) phase and NaPA-rich (**bottom**) phase, determined for the systems composed by PEG 600 + NaPA 8000 + water + 10 wt% of electrolytes and without electrolytes as a control.

Figure 5 depicts a schematic representation of the ion partition and related phenomena. As expected, the ABS coexisting phases maintain their electroneutrality; however, the addition of electrolytes can induce a dramatic effect on the phase equilibria, particularly in the compartmentalization mechanism of the NaPA 8000 counterions [11]. For instance, if a specific ion moves from the PEG-rich phase to the NaPA-rich phase, a counter-ion must be transferred in the opposite direction in order to maintain the electroneutrality in the phases [34,35]. Therefore, when the IL electrolyte is only partially concentrated in the PEG-rich phase with a significant part of it in the NaPA-rich phase, as happens with [Ch][DHcit] and [Ch][Bit], there is a partition of Na^+^ into the PEG-rich phase (Figure 5). By contrast, when an electrolyte extensively partitions into the PEG-rich phase, as [Ch]Cl and [Ch][Ac] (stronger salting-in agents), there is no significant Na^+^ migration to the opposite phase (Figure 5). This effect can be confirmed by the observed differences between the partition coefficients of sodium cation (*Kp* Na^+^) shown in Figure 6. The *Kp* Na^+^ was calculated by the ratio of the concentrations of Na^+^ in the PEG-rich phase to that in the NaPA-rich phase, determined using a sodium ion-selective electrode (additional data are given in the Supplementary Material—Table S25).

The logarithmic values of sodium cation’s partition coefficients [log (*Kp* Na^+^)] (Figure 6) of NaCl/Na[Ac]-added ABS are less negative than those without any electrolyte added, since the added electrolytes mainly partition to the top(PEG-rich) phase, allowing this phase to also contain sodium cations. Comparing the salt-based enhancers (NaCl/Na[Ac]) with the [Ch]-based enhancers ([Ch]Cl/[Ch][Ac]), it can be concluded that log (*Kp* Na^+^) is more negative in the latter, since in these, there are no extra sodium cations in added electrolytes, and thus only the contribution of the NaPA present in the bottom phase is the Na^+^. When [Ch]-based depressants ([Ch][DHcit]/[Ch][Bit]) are added as electrolytes, log (*Kp* Na^+^) is less negative than those when [Ch]-based enhancers ([Ch]Cl/[Ch][Ac]) are added. In the first two systems, there is a delocalization of the polyelectrolyte (NaPA) from the bottom towards the upper phase in order to maintain the electronegativity of the phases, since the added electrolytes, i.e., [Ch][DHcit] and [Ch][Bit], seem to have lower affinity to the top (PEG-rich) phase. It is important to note that these two ILs have stronger salting-out abilities (due to the more hydrophobic character of the anion), decreasing their interaction with the water and, consequently, with lower partition to the more hydrophilic top(PEG-rich) phase. Interestingly, the effect of each [Ch]-based IL is highly dependent of the relative partition of the electrolyte in the PEG-rich phase and their relative hydrophilicity degree. Therefore, due to favorable interactions (of hydrophilic nature), the more hydrophilic [Ch]Cl and [Ch][Ac] ILs (salting-in effect) are preferentially partitioned in the PEG-rich phase acting as ABS enhancers. To obtain further insights regarding the interactions occurring in each aqueous phase, vibrational studies by ATR-FTIR were performed. FTIR spectra of the top (PEG-rich) and bottom (NaPA-rich) phases of all ABS for systems with a composition of 25 wt% PEG 600 + 7.5 wt% NaPA 8000 + 10 wt% of electrolyte + 57.5 wt% water (the system with 67.5 wt% of water without electrolytes was used as a reference) were acquired. The ATR-FTIR spectra of the top phases (*cf.* Figure S6 in the Supplementary Material) of the studied ABS are very similar, showing only small differences in the absorbance intensity related with the higher or lower presence of the added electrolytes for systems containing ABS formation enhancers (NaCl, Na[Ac], [Ch]Cl and [Ch][Ac]) or ABS formation depressants ([Ch][DHcit] and [Ch][Bit). The intensity of these peaks corresponding to each IL anion is in agreement with the IL partition coefficients provided in Figure 3 and Figure 4, determined by ^1^H NMR and chloride ion-selective electrodes.

To investigate the possible interactions occurring between electrolytes and polymers, and consequently on the ABS phase formation mechanisms, we compared the spectra of the top (PEG-rich) and bottom (NaPA-rich) phases for ABS with each electrolyte with the spectrum of the ABS without electrolytes. The expanded ATR-FTIR spectra of main characteristic absorption bands are given in Figure S7 in the Supplementary Material. In the top phase, deviations in the main peaks corresponding to PEG were analysed, namely: the CH_2_ twisting at 960 cm^−1^; C–O, C–C stretching at 1097 cm^−1^, CH_2_ twisting at 1241 and 1278 cm^−1^ and CH_2_ wagging at 1341 cm^−1^ (peak assignment based on Shameli et al. [36]). Interestingly, there is no difference in the chemical shifts of the FTIR spectra of ABS without electrolytes and those with ILs/salts, showing that no major interactions between these and PEG are occurring in the water-rich systems studied. On the other hand, the comparative analysis of the expanded spectra of the bottom phases (NaPA-rich phase), particularly of the two bands occurring in the 1550–1600 cm^−1^ and 1400–1350 cm^−1^ that are typical for symmetrical stretching vibrations of carboxyl anions –COO^−^ [37,38], indicates some changes in the chemical shifts of these peaks, which are indicative of interactions occurring between NaPA and each IL/salt. The band at 1551 cm^−1^ does not change for the ABS without electrolyte and with electrolytes that act as ABS formation enhancers (NaCl, Na[Ac], [Ch]Cl and [Ch][Ac]); however, this band is slightly shifted (blueshift) to 1560 cm^−1^ for the systems using electrolytes acting as ABS formation depressants, namely [Ch][DHcit] and [Ch][Bit]. The interaction that causes the blueshift in the carboxyl anions absorption peak in the systems with ABS formation depressants also leads to a slight decrease toward the redshift in the band absorption at 1400 cm^−1^. These changes (marked with dashed red lines in Figure S7 in the Supplementary Material) confirm that [Ch][DHcit] or [Ch][Bit] establish favourable interactions with the carbonyl group of the NaPA polymer (since there is a decrease in the carboxylate group that remain as a free ion). The intensity of the chemical shift deviations in the PEG-rich phase additionally follows the IL partition coefficient and ABS phase-formation impact. Overall, these results show that interaction between NaPA and ILs plays a major role in ruling the ABS formation ability.

In summary, electrolytes acting as depressants ([Ch][DHcit] and [Ch][Bit]) in the ABS formation have demonstrated a lower affinity to the PEG-rich phase. Therefore, they tend to increase the entropy of mixing of the polyelectrolyte counterions and consequently decrease the ABS liquid–liquid demixing ability. This negative influence over the phase equilibria was here observed with organic electrolytes, as [Ch]-based ILs, being more evident when electrolytes with a lower affinity to the PEG-rich phase are used.

## 3. Materials and Methods

### 3.1. Materials

Poly(ethylene glycol) of molecular weights of 600, 2000, 4000 and 6000 g·mol^−1^ and the aqueous solution of sodium poly(acrylate) of 8000 g·mol^−1^ (45 wt%) were purchased from Sigma-Aldrich (St. Louis, MO, USA). Cholinium chloride, [Ch]Cl, and cholinium dihydrogencitrate, [Ch][DHcit], were acquired from Sigma-Aldrich; cholinium acetate, [Ch][Ac], and cholinium dihydrogenphosphate, [Ch][DHP], were obtained from Iolitec (Heilbronn, Germany); cholinium bitartrate, [Ch][Bit], was purchased from Acros Organics (Geel, Belgium). All ILs have a purity level higher than 98 wt%. All other chemicals were of reagent grade.

### 3.2. Determination of Phase Diagrams

Aqueous solutions of each phase-forming compound (PEG and NaPA) were prepared with a known concentration of each [Ch]-based IL and used to determine the respective binodal curves. The water samples used also contained each IL at the desired concentration to keep it constant along all the phase diagrams. The phase diagrams were determined by the cloud point titration method at 300.15 (±1) K and atmospheric pressure, according to previously described procedures [5,39]. The temperature was maintained by means of a double-jacketed cell coupled to a water bath circulator. The ternary system compositions were calculated by the weight quantification of all components added within ±10^−7^ kg. The detailed experimental weight fraction data are reported in Tables S1–S14.

The experimental binodal curves of each ABS were correlated using the following equation [27]:

(1) $$Y=A\exp\left(BX^{0.5}-CX^{3}\right),$$

where *A*, *B* and *C* are correlation constants, and *X* and *Y* are the weight concentrations (wt%) of NaPA 8000 and PEG 600, respectively. The respective constants and correlation coefficients for each system are reported in Tables S15–S20.

### 3.3. Quantification of the Cholinium Cation in the ABS Coexisting Phases

A mixture point within the biphasic region was selected, and an ABS was prepared by weighting the appropriate amounts of each compound: 25 wt% of PEG 600 + 7.5 wt% of NaPA 8000 + 10 wt% of each [Ch]-based IL + water. After mixing all components, the systems were allowed to equilibrate for 16 h, followed by centrifugation at 1100 rpm for 10 min. The phases were carefully separated and collected for the measurement of their volumes, weight, and further quantification of each electrolyte’s cation/anion at the coexisting phase by ^1^H NMR spectroscopy. The amount of cholinium cation ([Ch]^+^) present in each phase was quantified using a Bruker Avance 300 spectrometer at 300.13 MHz, whereas an NMR tube containing 500 μL of solvent plus the internal standard (dimethyl sulfoxide, DMSO, + 10% *v/v* benzene) and 100 μL of the ABS phase within a neat tube (to avoid precipitation of the NaPA present in the samples by DMSO) was prepared gravimetrically with an associated uncertainty of ±10^−7^ kg. By quantifying the moles of [Ch]^+^ present in each phase by means of the benzene internal standard, the concentration of [Ch]^+^ was then determined.

### 3.4. Quantification of Sodium and Chloride Ions in the ABS Coexisting Phases

The quantification of the sodium cation (Na^+^) and chloride anion (Cl^−^) present in each phase of different ABS was determined using Metrohm ion-selective electrodes (of sodium or chloride, respectively) in a Metrohm 904 Titrando titrator according to the manufacturer’s instructions, whereas the concentration of those ions was given. The limit of detection for sodium- and chloride-selective electrodes are 5 × 10^−6^ and 1 × 10^−5^ mol·L^−1^, respectively.

### 3.5. Determination of Partition Coefficients of Electrolytes (Cations and Anions) Present in ABS

*Kp* [Ch]^+^/ Na^+^/Cl^−^ are defined as the ratio between the concentration of each electrolyte ([Ch]^+^, Na^+^ and Cl^−^) present in the PEG-rich phase (top) and NaPA-rich phase (bottom), as described by the following equation (for [Ch]^+^, as example):

(2) $$K_{p\;}{\left[\mathrm{Ch}\right]}^{+}=\frac{{\left[\mathrm{Ch}\right]}^{+}{}_{\mathrm{PEG}}}{{\left[\mathrm{Ch}\right]}^{+}{}_{\mathrm{NaPA}}},$$

where ${\left[\mathrm{Ch}\right]}^{+}{}_{\mathrm{PEG}}$ and ${\left[\mathrm{Ch}\right]}^{+}{}_{\mathrm{NaPA}}$ are the concentration of [Ch]^+^ present in the PEG- and NaPA-rich phases, respectively.

Regarding the partition coefficients for the bitartrate ([Bit]^−^), acetate ([Ac]^−^) and dihydrogencitrate ([DHcit]^−^) anions present in the respective ABS, their determination was performed upon the calculation of the relationship between their characteristic ^1^H NMR spectra peak from each top (PEG)-and bottom (NaPA)-rich phases.

### 3.6. Vibrational Studies of Electrolytes Measured by ATR-FTIR in the ABS Coexisting Phases

After ABS preparation according to Section 3.3 (25 wt% of PEG 600 + 7.5 wt% of NaPA 8000 + 10 wt% of each electrolyte + water), the top and bottom phases of each ABS were carefully separated and directly used for attenuated total reflection Fourier-transform infrared spectroscopy (ATR-FTIR). The ATR-FTIR spectra of each ABS phase were recorded on a Bruker Tensor 27 spectrometer. All spectra measurements were acquired in the range 4000–350 cm^−1^, averaging 256 scans per sample and at a resolution of 4 cm^−1^.

## 4. Conclusions

Opposite trends in the PEG-NaPA ABS formation ability were herein reported for the first time using [Ch]-based ILs as electrolytes. ILs that extensively partition to the PEG-rich phase act as inorganic salts, enhancing the two-phase formation ability, while those that have a lower partition to the PEG-rich phase decrease the ABS formation capacity. The distribution of the electrolyte ions between the coexisting phases and the polyelectrolyte ion compartmentalization are key factors ruling the formation of PEG-NaPA-based ABS. The use of organic salts/ILs as electrolytes allows a wider diversity of phase equilibria, as well as the manipulation of their potential to improve the selectivity and efficiency of separation processes.

## Data Availability

Not applicable.

## Supplementary Materials

The following are available online, Figure S1: Binodal curves for the systems formed by PEG (different molecular weight) + NaPA 8000 + water + 5 wt% of [Ch]^+^-IL as electrolyte.; Figure S2: Binodal curves for the ABS formed by PEG (different molecular weight) + NaPA 8000 + water + 5 wt% of [Ch]Cl as electrolyte.; Figure S3: Binodal curves, at 300.15 K, for ABS composed of PEG 600, NaPA 8000 and each [Ch]-based IL used as electrolyte.; Figure S4: Binodal curves, at 300.15 K, for ABS composed of PEG 600, NaPA 8000, and NaCl used as electrolyte.; Figure S5: Binodal curves, at 300.15 K, for ABS composed of PEG 600, NaPA 8000, and NaAcetate used as electrolyte; Figure S6. ATR-FTIR spectra of the top (PEG-rich) phases (A) and bottom (NaPA-rich) phases (B) measured for the ABS composed of 25 wt% of PEG 600 + 7.5 wt% NaPA 8000 + water (67.5 wt% system without electrolyte or 57.5 wt% with electrolyte) + 10 wt% of the respective electrolyte.; Figure S7. ATR-FTIR expanded spectra of the top (PEG-rich) phases (A) and bottom (NaPA-rich) phases (B) measured for the ABS composed of 25 wt% of PEG 600 + 7.5 wt% NaPA 8000 + water (67.5 wt% system without electrolyte or 57.5 wt% with electrolyte) + 10 wt% of the respective electrolyte.; Table S1: Experimental binodal weight fraction data for the systems composed of PEG (1) + NaPA 8000 (2) + 5 wt% [Ch]Cl + water, determined at 300.15 K.; Table S2: Experimental binodal weight fraction data for the systems composed of PEG (1) + NaPA 8000 (2) + 5 wt% [Ch][Ac] + water, determined at 300.15 K.; Table S3: Experimental binodal weight fraction data for the systems composed of PEG (1) + NaPA 8000 (2) + 5 wt% [Ch][DHP] + water, determined at 300.15 K.; Table S4: Experimental binodal weight fraction data for the systems composed of PEG (1) + NaPA 8000 (2) + 5 wt% [Ch][DHcit] + water, determined at 300.15 K.; Table S5: Experimental binodal weight fraction data for the systems composed of PEG (1) + NaPA 8000 (2) + 5 wt% [Ch][Bit] + water, determined at 300.15 K.; Table S6: Experimental binodal weight fraction data for the systems composed of PEG 600 (1) + NaPA 8000 (2) + water + 1 wt% of [Ch]X, X = Cl and [Ac], determined at 300.15 K.; Table S7: Experimental binodal weight fraction data for the systems composed of PEG 600 (1) + NaPA 8000 (2) + water + 1 wt% of [Ch]X, X = [DHP], [DHcit] and [Bit], determined at 300.15 K.; Table S8: Experimental binodal weight fraction data for the systems composed of PEG 600 (1) + NaPA 8000 (2) + water + 2.5 wt% of [Ch]X, X = Cl and [Ac], determined at 300.15 K.; Table S9: Experimental binodal weight fraction data for the systems composed of PEG 600 (1) + NaPA 8000 (2) + water + 2.5 wt% of [Ch]X, X = [DHP], [Bit] and [DHcit], determined at 300.15 K.; Table S10: Experimental binodal weight fraction data for the systems composed of PEG 600 (1) + NaPA 8000 (2) + water + 5 wt% of [Ch]X, X = Cl and [Ac], determined at 300.15 K.; Table S11: Experimental binodal weight fraction data for the systems composed of PEG 600 (1) + NaPA 8000 (2) + water + 5 wt% of [Ch]X, X = [DHP], [DHcit] and [Bit], determined at 300.15 K.; Table S12: Experimental binodal weight fraction data for the systems composed of PEG 600 (1) + NaPA 8000 (2) + water + 10 wt% of [Ch]X, X = Cl and [Ac], determined at 300.15 K.; Table S13: Experimental binodal weight fraction data for the systems composed of PEG 600 (1) + NaPA 8000 (2) + water + 10 wt% of [Ch]X, X = [DHP], [DHcit] and [Bit], determined at 300.15 K.; Table S14: Experimental binodal weight fraction data for the systems composed of PEG 600 (1) + NaPA 8000 (2) + water, determined at 300.15 K.; Table S15: Experimental binodal weight fraction data for the systems composed of PEG 600 (1) + NaPA 8000 (2) + X wt% NaCl + water, determined at 300.15 K.; Table S16: Experimental binodal weight fraction data for the systems composed of PEG 600 (1) + NaPA 8000 (2) + X wt% NaOAc + water, determined at 300.15 K.; Table S17: Correlation parameters to describe the binodal curve, for the system composed of PEG 600 + NaPA 8000.; Table S18: Correlation parameters to describe the binodal curves, for the systems composed of PEG 600 + NaPA 8000 + [Ch]Cl.; Table S19: Correlation parameters to describe the binodal curves, for the systems composed of PEG 600 + NaPA 8000 + [Ch][Ac].; Table S20: Correlation parameters to describe the binodal curves, for the systems composed of PEG 600 + NaPA 8000 + [Ch][DHP].; Table S21: Correlation parameters to describe the binodal curves, for the systems composed of PEG 600 + NaPA 8000 + [Ch][DHcit].; Table S22: Correlation parameters to describe the binodal curves, for the systems composed of PEG 600 + NaPA 8000 + [Ch][Bit].; Table S23: Number of moles of [Ch]^+^ at each phase, the volume of the phase and *Kp* values of [Ch]^+^.; Table S24: Concentration of chloride anion at each corresponding phase, the absolute values of the integral of the ^1^H RNM peaks of the anions and *Kp* values of the anion for each electrolyte in the ABS.; Table S25: Concentration of sodium cation at each corresponding phase, *Kp* and log *Kp* values of sodium cation in studied ABSs.

## Sample Availability

Samples of the compounds are not available from the authors.
