# Supplementary material for: Opposite Effects Induced by Cholinium-Based Ionic Liquid Electrolytes in the Formation of Aqueous Biphasic Systems Comprising Polyethylene Glycol and Sodium Polyacrylate
Source: Molecules. 2021 Oct 31;26(21):6612. doi: 10.3390/molecules26216612 (PMC8587793; doi:10.3390/molecules26216612)
Supplement: Supplementary file 1 [file molecules-26-06612-s001.zip › molecules-1321483-supplementary.pdf]

molecules

MDPI

Supplementary Materials

Opposite effects Induced by Cholinium-Based Ionic Liquid Electrolytes in the Formation of Aqueous Biphasic Systems Comprising Polyethylene Glycol and Sodium Polyacrylate

Sandra C. Bernardo [1], Emanuel V. Capela [2], Jorge F. B. Pereira [3], Sónia P. M. Ventura [2], Mara G. Freire [2,*] and João A. P. Coutinho [2,*]

[1] CICS-UBI–Health Sciences Research Centre, University of Beira Interior, 6200-506 Covilhã, Portugal; sandracsbernardo@gmail.com

[2] CICECO–Aveiro Institute of Materials, Department of Chemistry, University of Aveiro, 3810-193 Aveiro, Portugal; emanuelcapela@ua.pt (E.V.C.); spventura@ua.pt (S.P.M.V.)

[3] Univ Coimbra, CIEPQPF, Department of Chemical Engineering, Rua Sílvio Lima, Pólo II–Pinhal de Marrocos, 3030-790 Coimbra, Portugal; jfbpereira@eq.uc.pt

* Correspondence: maragfreire@ua.pt (M.G.F.); jcoutinho@ua.pt (J.A.P.C.); Tel: +351-234370200 (M.G.F. & J.A.P.C.); Fax: +351-234370084 (M.G.F. & J.A.P.C.)

Effect of the PEG molecular weight in the formation of ABS composed of PEG + NaPA 8000 + water + 5 wt% of [Ch]⁺-IL as electrolyte

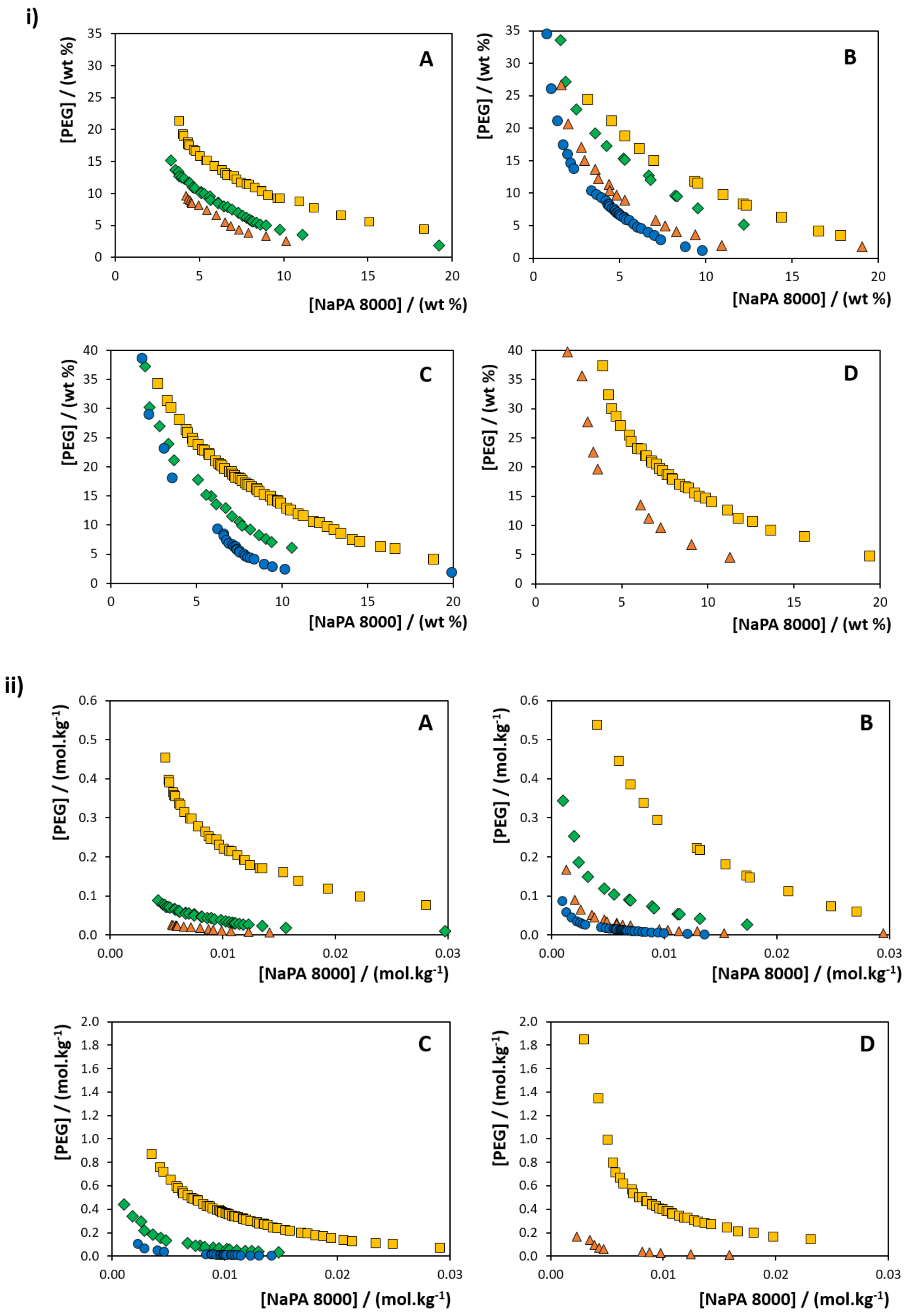

Figure S1. Binodal curves for the systems formed by PEG (different molecular weight) + NaPA 8000 + water + 5 wt% of [Ch]⁺-IL as electrolyte: [Ch][Ac] (A), [Ch][DHP] (B), [Ch][DHcit] (C) and [Ch][Bit] (D). PEG molecular weight: PEG 600 (■); PEG 2000 (▲); PEG 4000 (◆); PEG 6000 (●).

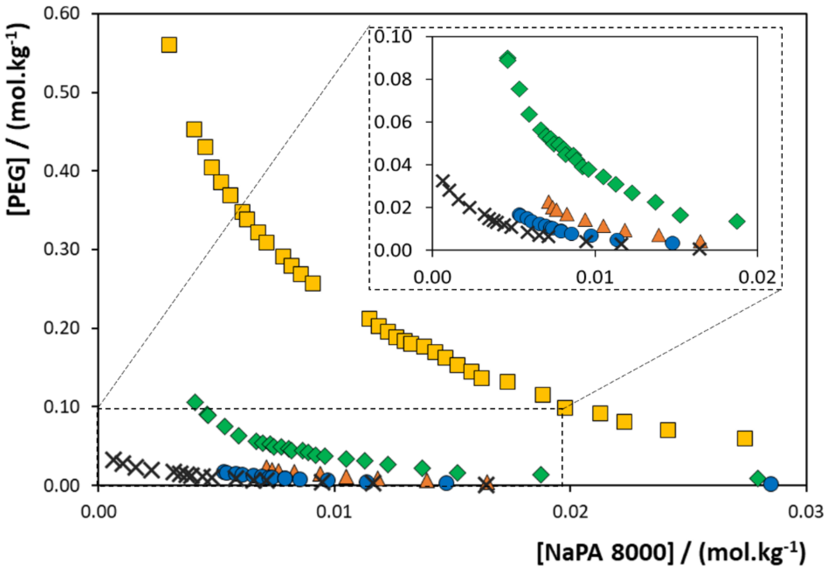

Figure S2. Binodal curves for the ABS formed by PEG (different molecular weight) + NaPA 8000 + water + 5 wt% of [Ch]Cl as electrolyte. PEG molecular weight: PEG 600 (■); PEG 2000 (▲); PEG 4000 (◆); PEG 6000 (●).

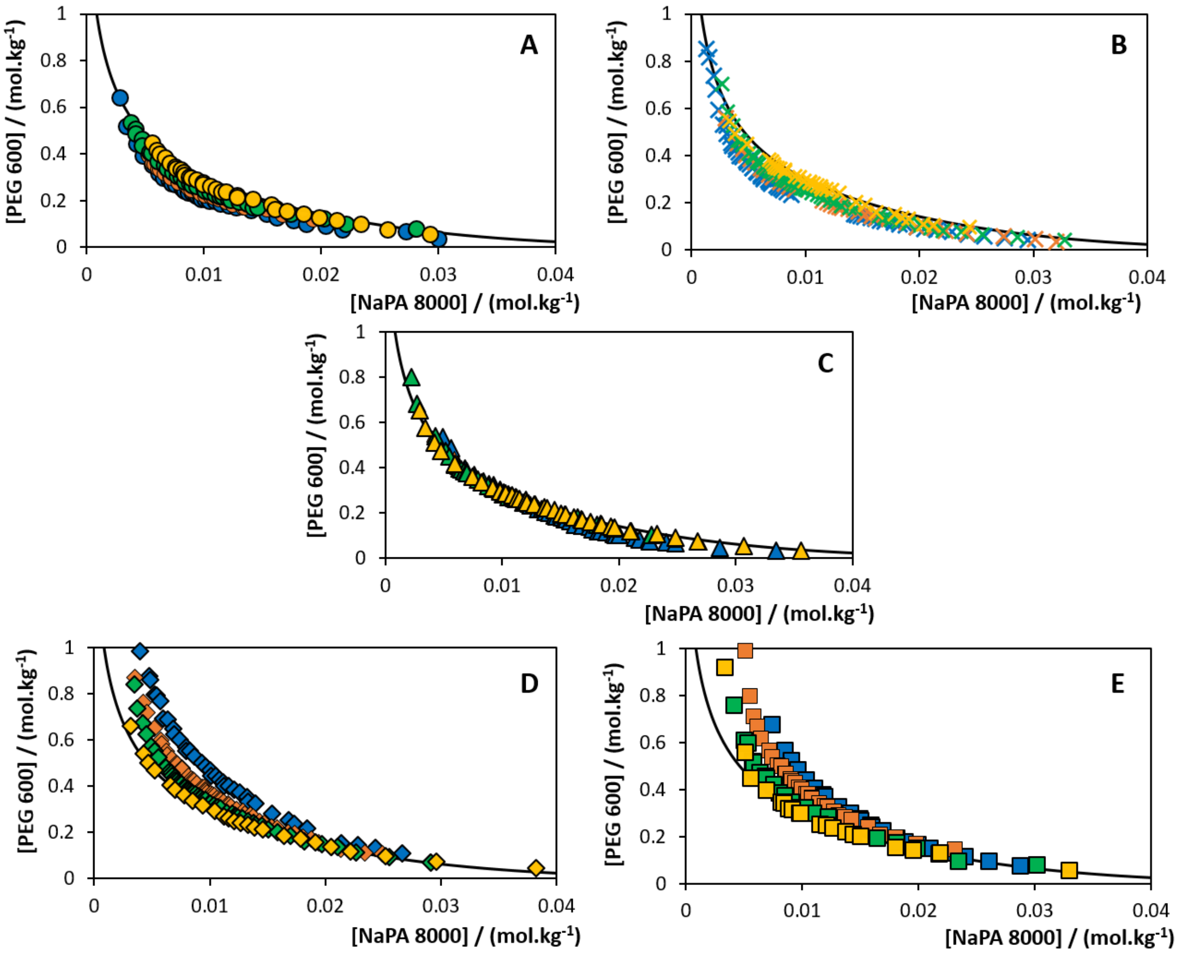

Figure S3. Binodal curves, at 300.15 K, for ABS composed of PEG 600, NaPA 8000, and each [Ch]+-based IL used as electrolyte: **a)** [Ch][Ac] (●); **b)** [Ch]Cl (✕); **c)** [Ch][DHP] (▲); **d)** [Ch][DHcit] (◆); and **e)** [Ch][Bit] (■). Distinct colours represent different concentrations, namely 10 wt% (**blue**), 5 wt% (**orange**), 2.5 wt% (**green**), 1 wt% (**yellow**). The ABS formed by PEG 600 and NaPA 8000 without electrolyte is represented by (—).

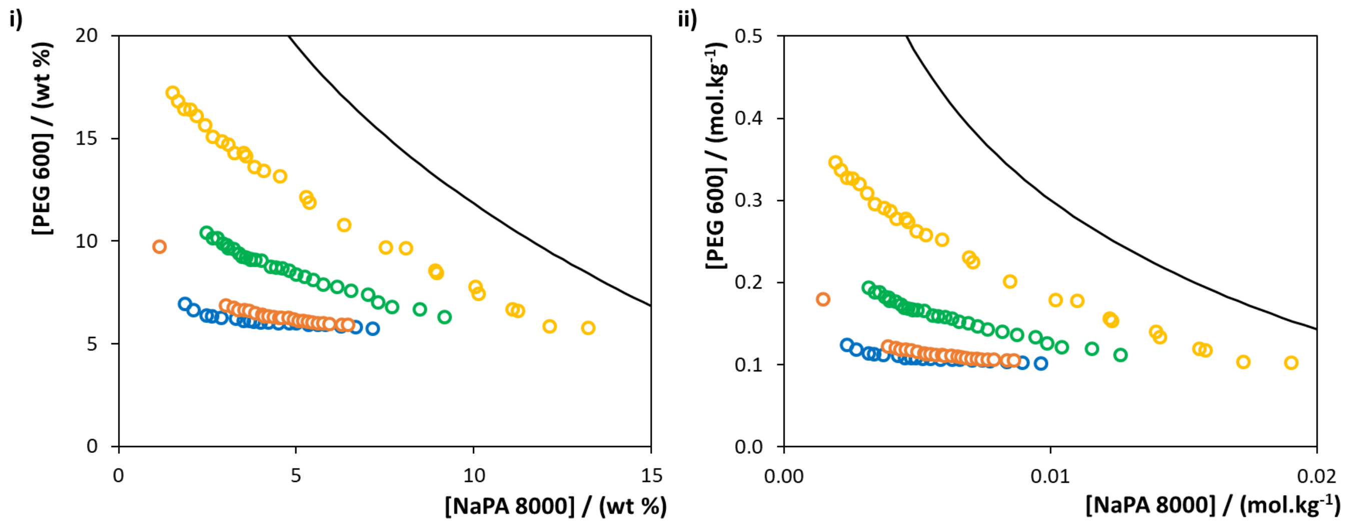

Figure S4. Binodal curves, at 300.15 K, for ABS composed of PEG 600, NaPA 8000, and NaCl used as electrolyte. Distinct colours represent different concentrations, namely 10 wt% (**blue**), 5 wt% (**orange**), 2.5 wt% (**green**), 1 wt% (**yellow**).

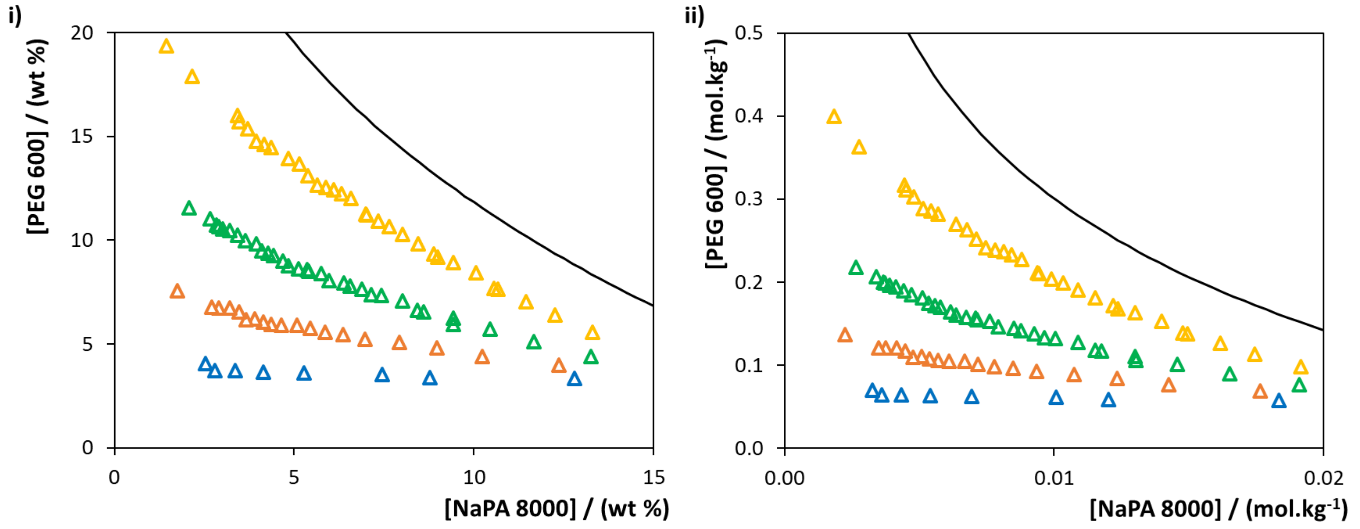

Figure S5. Binodal curves, at 300.15 K, for ABS composed of PEG 600, NaPA 8000, and NaAcetate used as electrolyte. Distinct colours represent different concentrations, namely 10 wt% (**blue**), 5 wt% (**orange**), 2.5 wt% (**green**), 1 wt% (**yellow**).

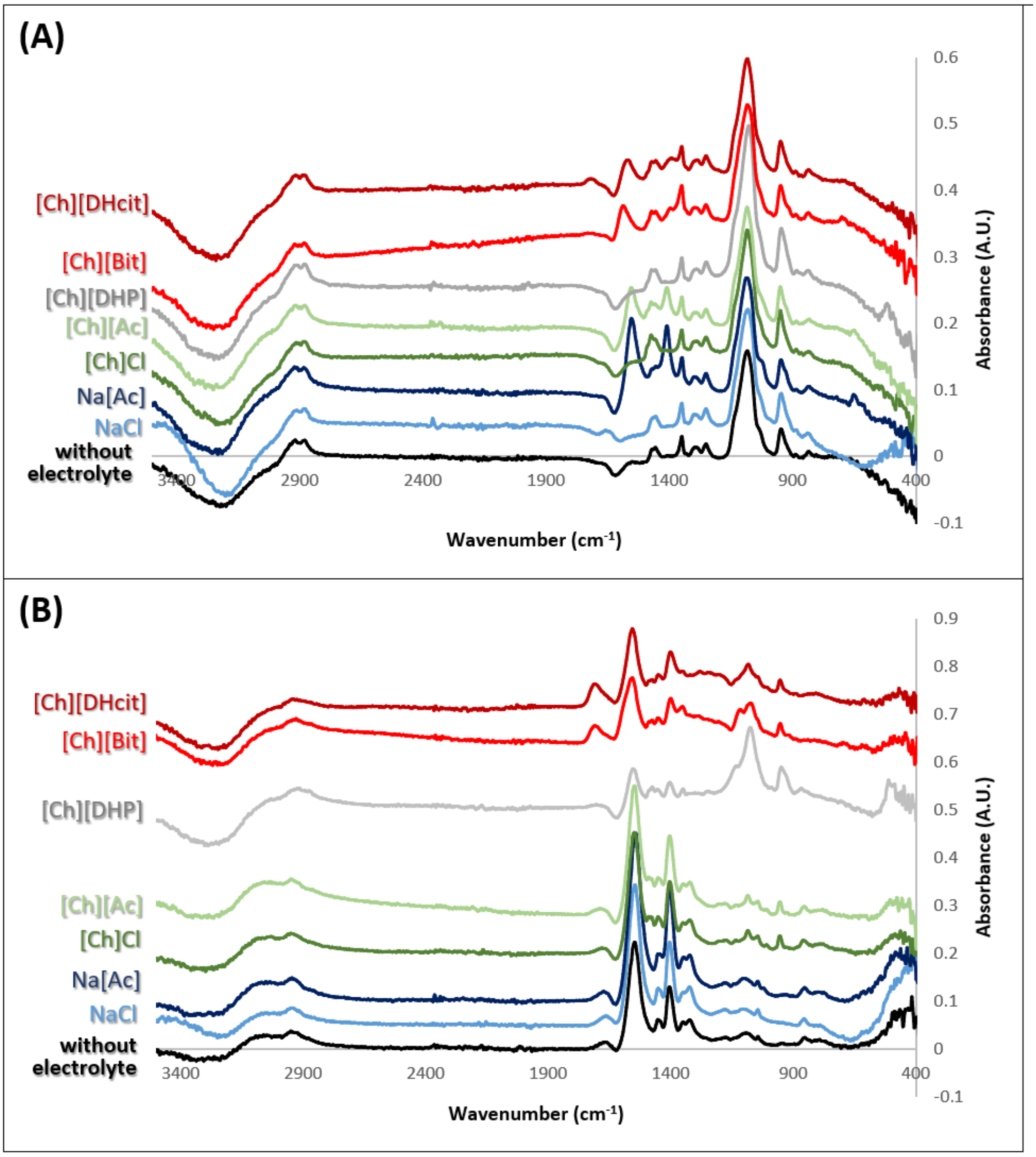

Figure S6. ATR-FTIR spectra of the top (PEG-rich) phases (A) and bottom (NaPA-rich) phases (B) measured for the ABS composed of 25 wt% of PEG 600 + 7.5 wt% NaPA 8000 + water (67.5 wt% system without electrolyte or 57.5 wt% with electrolyte) + 10 wt% of the respective electrolyte.

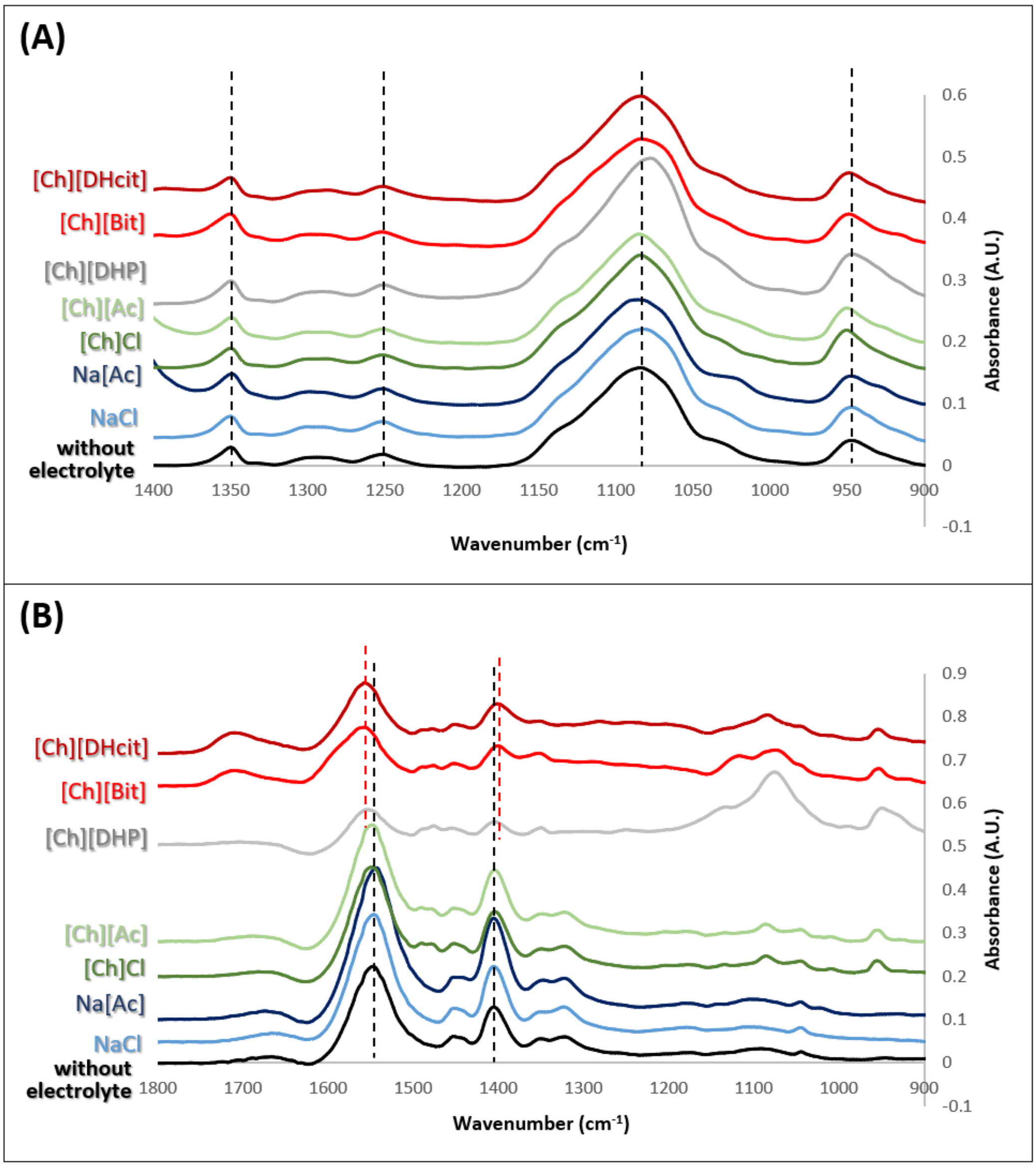

Figure S7. ATR-FTIR expanded spectra of the top (PEG-rich) phases (A) and bottom (NaPA-rich) phases (B) measured for the ABS composed of 25 wt% of PEG 600 + 7.5 wt% NaPA 8000 + water (67.5 wt% system without electrolyte or 57.5 wt% with electrolyte) + 10 wt% of the respective electrolyte.

Experimental weight fraction data corresponding to the solubility curves

Table S1. Experimental binodal weight fraction data for the systems composed of PEG (1) + NaPA 8000 (2) + 5 wt% [Ch]Cl + water, determined at 300.15K.

PEG 600		PEG 2000		PEG 4000		PEG 6000	
100 w_1	100 w_2	100 w_1	100 w_2	100 w_1	100 w_2	100 w_1	100 w_2
25.1491	2.3364	17.3944	3.1726	8.3313	5.3989	9.1430	4.0836
21.3523	3.1556	15.2522	3.5561	7.4996	5.5688	8.8037	4.1666
20.5117	3.5010	15.0750	3.5767	7.0830	5.7551	8.2269	4.4431
19.5203	3.7108	13.0980	4.1009	6.3609	6.2144	7.5748	4.6539
18.7926	4.0016	11.2728	4.5327	5.4744	6.9948	6.9902	5.0067
18.1432	4.2878	10.1529	5.0678	4.4121	7.7595	6.5283	5.2529
17.2884	4.6484	9.6631	5.2854	3.6436	8.6418	6.0622	5.4773
16.8672	4.7913	9.4591	5.5026	2.7929	10.0172	5.8532	5.5693
16.1764	5.1497	9.0366	5.6252	1.7434	11.6509	5.1807	5.9555
15.6390	5.4029	8.9884	5.8491			5.0350	5.9559
14.8483	5.8827	8.6352	6.0549			4.4882	6.4069
14.3791	6.1344	8.2402	6.1377			3.9017	7.2251
13.8735	6.4168	8.1707	6.4707			2.7645	8.3239
13.3383	6.7710	7.7865	6.6300			2.0052	10.5569
11.2692	8.4250	7.2584	6.8632			0.9007	18.5574
10.8306	8.6766	7.0430	7.1218				
10.5197	8.9421	6.4155	7.7440				
10.1831	9.1599	5.8331	8.2823				
9.9022	9.3963	5.1084	8.9478				
9.7855	9.5786	4.2865	9.8825				
9.5854	9.9409	3.1842	10.8559				
9.2636	10.2435	2.6150	13.0353				
8.8711	10.5180	1.8522	18.2685				
8.4300	10.8399						
7.9695	11.2180						
7.5918	11.4997						
7.3320	12.1887						
6.4983	13.1027						
5.6207	13.6698						
5.2406	14.5508						
4.6566	15.1248						
4.0526	16.1773						
3.4361	17.9787						
2.6852	19.4121						
1.9918	20.3835						

Table S2. Experimental binodal weight fraction data for the systems composed of PEG (1) + NaPA 8000 (2) + 5 wt% [Ch][Ac] + water, determined at 300.15K.

PEG 600		PEG 2000		PEG 4000	
100 *w1*	100 *w2*	100 *w1*	100 *w2*	100 *w1*	100 *w2*
21.4017	3.7864	15.1460	3.3006	9.6174	4.2166
19.2553	3.9999	13.6992	3.5856	9.1064	4.2968
18.9732	4.0378	13.4232	3.7430	8.9808	4.4429
17.9705	4.3047	12.6516	3.8075	8.6367	4.5057
17.7180	4.3110	12.4560	3.9420	8.4873	4.5405
17.5451	4.4039	12.2705	4.0911	8.1821	4.9635
16.7975	4.6627	11.6826	4.3879	7.3844	5.4372
16.6444	4.7474	11.5988	4.4120	6.5967	6.0116
15.8893	5.0285	10.8861	4.6215	5.5113	6.5327
15.1910	5.3814	10.8032	4.7175	4.9028	6.8400
15.1512	5.4554	10.1876	5.0888	4.3457	7.3458
14.3308	5.8885	10.0487	5.0911	3.8404	7.8922
14.3043	5.8977	9.9626	5.3045	3.2958	8.9568
13.7125	6.3169	9.5534	5.6218	2.5007	10.1638
13.1869	6.5189	8.9641	5.6489		
12.8489	6.6510	8.5555	6.0932		
12.7660	7.0384	8.4145	6.1125		
12.1765	7.1943	7.9354	6.4491		
11.7017	7.4732	7.8962	6.6856		
11.5019	7.7788	7.4211	6.9153		
11.3975	7.9332	7.0224	7.2999		
10.9419	8.2892	6.4482	7.5787		
10.3918	8.6923	6.1606	7.7734		
10.3231	8.7050	5.8875	7.9909		
9.7496	9.0447	5.6539	8.1069		
9.2713	9.6181	5.5980	8.1839		
9.2632	9.7591	5.3625	8.4008		
8.7851	10.9494	5.1170	8.6253		
7.7252	11.8046	4.9913	8.9642		
6.6225	13.3984	4.3138	9.7724		
5.5533	15.0832	3.5596	11.1107		
4.3823	18.3445	1.8910	19.2213		

Table S3. Experimental binodal weight fraction data for the systems composed of PEG (1) + NaPA 8000 (2) + 5 wt% [Ch][DHP] + water, determined at 300.15K.

PEG 600		PEG 2000		PEG 4000		PEG 6000			
100 *w1*	100 *w2*	100 *w1*	100 *w2*	100 *w1*	100 *w2*	100 *w1*	100 *w2*	100 *w1*	100 *w2*
24.4297	3.1373	40.6956	0.8139	40.1585	1.0337	34.4945	0.7568	6.9796	4.8275
21.0999	4.5286	33.5871	1.5930	26.6726	1.6020	26.0814	1.0361	6.9490	4.8923
18.8096	5.3104	27.1566	1.8711	20.6659	2.0199	21.1803	1.4024	6.7227	4.9847
16.8888	6.1427	22.9208	2.4979	17.1142	2.7721	17.4782	1.7365	6.7185	5.0277
15.0411	6.9715	19.1875	3.5885	15.0704	2.9723	16.0258	1.9903	6.5236	5.0809
11.7951	9.3382	17.2279	4.2429	13.6400	3.5964	14.6184	2.1740	6.3523	5.2549
11.5536	9.5282	15.3528	5.2193	12.2534	3.7621	13.7749	2.3465	6.2266	5.2721
9.8168	10.9834	15.0861	5.3178	11.3541	4.4049	10.3681	3.3771	5.9715	5.3706
8.3572	12.1637	12.7565	6.6735	10.3885	4.4326	9.8122	3.6758	5.7921	5.5544
8.1333	12.3493	12.0768	6.7858	9.7035	4.8143	9.2789	3.9486	5.2872	5.7989
6.2687	14.3867	9.6191	8.2111	8.9501	5.3091	8.8003	4.1911	4.7896	6.0468
4.2163	16.5466	9.5248	8.3384	5.8503	7.0902	8.2964	4.3033	4.5827	6.2645
3.4545	17.8155	7.6681	9.5471	4.9581	7.6451	8.2094	4.3717	4.0318	6.6381
		5.1469	12.1886	4.0733	8.2967	7.9321	4.4122	3.5199	7.0101
				3.6442	9.3823	7.9146	4.4346	2.8077	7.3688
				1.9809	10.9239	7.6513	4.5819	1.7734	8.8212
				1.7982	19.0826	7.5988	4.6636	1.1364	9.7894
						7.3190	4.7252	1.1345	20.1040
						7.2579	4.7600		

Table S4. Experimental binodal weight fraction data for the systems composed of PEG (1) + NaPA 8000 (2) + 5 wt% [Ch][DHcit] + water, determined at 300.15K.

PEG 600				PEG 2000		PEG 6000			
100 *w1*	100 *w2*	100 *w1*	100 *w2*	100 *w1*	100 *w2*	100 *w1*	100 *w2*	100 *w1*	100 *w2*
57.6120	0.7478	17.2403	7.8416	46.9251	0.8473	38.6150	1.8036	5.3881	7.5172
34.3054	2.7416	17.0602	7.9706	40.6456	1.4423	29.0086	2.2189	5.0224	7.7864
31.3698	3.2824	16.8770	8.0700	37.1967	2.0046	23.1217	3.1035	4.7198	7.8390
30.1640	3.4983	16.6071	8.2083	30.2088	2.2366	18.1094	3.5725	4.4872	7.9944
28.0897	3.9969	16.2660	8.4998	26.9246	2.8231	9.3457	6.2399	4.3237	8.1565
26.4449	4.3812	16.0804	8.5396	23.9236	3.3458	8.5274	6.5801	4.1221	8.3500
25.9101	4.4693	15.6927	8.6625	21.1660	3.6994	8.1735	6.6038	3.2682	8.9434
24.8802	4.7702	15.2476	8.9554	17.8206	5.0826	7.4045	6.7464	2.8488	9.4386
24.3640	4.8088	14.9280	9.3237	14.9694	5.8651	6.8973	6.8790	2.4648	10.1561
23.7758	5.0868	14.3502	9.4292	12.8949	6.7083	6.6356	7.1129	1.8362	19.9296
22.9845	5.3585	14.2403	9.7241	11.4689	7.0586	6.3505	7.1843		
22.8497	5.4682	14.0816	9.7553	10.5672	7.5269	6.2807	7.2981		
22.2941	5.7055	13.7589	9.9232	9.9017	7.6790	5.8581	7.3242		
22.1434	5.7341	12.9219	10.2577	9.2061	8.1421	5.7508	7.3802		
21.0172	6.0988	12.6329	10.4988	8.3119	8.6505	5.5907	7.4979		
20.5719	6.3000	11.8999	10.9395	7.5862	9.0439				
20.3958	6.4215	11.5913	11.2237	7.0916	9.4050				
20.1778	6.4756	10.6640	11.8196	6.0671	10.5600				
19.7313	6.6378	10.4120	12.1759	13.5170	6.1651				
19.1847	6.9432	9.7228	12.6220	15.1955	5.5796				
19.1301	7.0480	9.2715	13.0348						
18.7089	7.1195	8.5963	13.4540						
18.5329	7.2034	7.5510	14.1189						
18.2212	7.2600	7.1311	14.5535						
18.0815	7.4733	6.3375	15.7635						
17.9678	7.5607	5.9758	16.6236						
17.6495	7.6487	4.1325	18.8669						

Table S5. Experimental binodal weight fraction data for the systems composed of PEG (1) + NaPA 8000 (2) + 5 wt% [Ch][Bit] + water, determined at 300.15K.

PEG 600				PEG 4000	
100 *w1*	100 *w2*	100 *w1*	100 *w2*	100 *w1*	100 *w2*
62.2136	1.7814	19.4539	7.3749	39.6989	1.8437
52.5733	2.3021	18.7232	7.6140	35.6251	2.6937
44.6771	3.2771	18.6921	7.7185	27.7177	3.0314
37.3126	3.8869	18.0215	7.9331	22.5812	3.3373
32.3674	4.2323	17.9061	7.9695	19.6730	3.6096
29.9647	4.4224	17.1094	8.3706	13.4993	6.0994
28.6764	4.6788	16.5921	8.6832	11.2195	6.5543
27.0796	4.9042	16.4422	8.8648	9.6106	7.2659
25.4448	5.4349	15.5315	9.2420	6.7214	9.0401
24.3991	5.5574	14.9597	9.4808	4.5749	11.2690
23.2431	5.9013	14.6365	9.8653	1.7670	20.0187
23.1088	6.1244	14.0050	10.2305		
21.9647	6.3742	12.6883	11.1395		
21.9000	6.4050	11.2239	11.7630		
21.0747	6.7205	10.6607	12.5969		
20.8167	6.7377	9.2180	13.6766		
20.4978	6.9869	8.0577	15.5889		
19.7306	7.1758	4.8035	19.3919		

Table S6. Experimental binodal weight fraction data for the systems composed of PEG 600 (1) + NaPA 8000 (2) + water + 1 wt% of [Ch]X, X = Cl and [Ac], determined at 300.15K.

[Ch]Cl				[Ch][Ac]			
100 *w1*	100 *w2*	100 *w1*	100 *w2*	100 *w1*	100 *w2*	100 *w1*	100 *w2*
24.7644	2.5866	8.7921	11.4887	21.1471	4.2748	8.0249	12.8621
22.8099	2.9850	8.2995	12.4953	20.0032	4.5362	7.2308	13.6659
21.1635	3.7729	7.6290	12.8927	19.4416	4.7412	6.4584	14.5580
18.6200	5.1626	7.3259	13.2123	18.7358	5.0813	5.6921	15.7508
18.3432	5.3492	6.2233	14.0118	17.9019	5.3799	4.4936	17.0376
17.9359	5.4424	5.8839	14.5126	17.1447	5.6854	3.2654	18.9647
17.4975	5.5353	5.4269	16.3113	16.8365	5.7414		
17.3001	5.7004			16.6972	5.9994		
17.1200	5.8521			16.2704	6.2119		
16.9294	6.1327			15.7760	6.2494		
16.6101	6.1703			15.3494	6.5435		
15.8423	6.7780			15.1687	6.6279		
15.5939	7.0285			14.8980	6.9343		
14.9019	7.3140			14.3730	7.3603		
14.7492	7.6450			13.9868	7.3612		
14.6913	7.6576			13.6478	7.7530		
14.3648	7.8848			13.1188	8.0730		
14.1696	8.0844			12.6152	8.3937		
13.9223	8.3110			12.5757	8.6880		
13.4772	8.3933			11.8770	9.2930		
13.4633	8.6620			11.4258	9.3214		
13.1352	8.9489			11.0650	10.1871		
12.7467	9.3719			9.8967	11.2003		
10.9479	10.3489			9.0564	11.3919		
10.2497	10.8720			8.4841	12.0351		

Table S7. Experimental binodal weight fraction data for the systems composed of PEG 600 (1) + NaPA 8000 (2) + water + 1 wt% of [Ch]X, X = [DHP], [DHcit] and [Bit], determined at 300.15K.

[Ch][DHP]				[Ch][DHcit]		[Ch][Bit]	
100 *w1*	100 *w2*	100 *w1*	100 *w2*	100 *w1*	100 *w2*	100 *w1*	100 *w2*
28.2076	2.2987	8.7364	12.3446	28.3673	2.4709	35.6599	2.5653
25.6730	2.6584	8.3535	12.8696	24.5402	3.2896	25.1579	3.8820
23.5209	3.2325	8.1968	12.8998	23.0655	3.5776	21.3774	4.2111
22.1828	3.6726	7.7622	13.3778	21.9428	4.0033	19.3431	5.2412
20.0503	4.5662	7.5309	13.5488	19.6006	5.0370	17.3022	6.1283
17.8177	5.6243	6.7364	14.3643	18.7389	5.2753	17.1258	6.3010
16.8300	6.1749	6.0453	15.6966	17.8160	5.8428	16.2485	6.5636
15.7126	6.8737	5.2533	16.5986	16.8660	6.3675	16.0653	6.8077
15.0580	7.2959	4.2589	17.6372	15.9030	7.0111	15.3889	7.2372
14.6884	7.5361	3.1925	19.7201	15.0135	7.7197	15.2781	7.2661
14.6338	7.5885	2.0471	22.1697	13.9400	8.2194	13.2115	8.4185
14.2320	7.9715			13.5506	8.5153	13.0044	8.7555
13.7331	8.2703			13.0627	8.8457	12.5520	9.1417
13.5107	8.4096			12.6685	9.1976	11.7446	9.8744
13.3123	8.7727			12.2728	9.6172	11.2502	10.3094
12.8512	8.8393			11.7206	9.9579	10.9512	10.6760
12.4411	9.2440			11.2720	10.4491	8.6116	12.5882
11.8839	9.8384			9.9805	11.5979	7.9057	13.4808
11.6682	9.9531			9.3224	12.4804	7.3917	14.8568
11.2657	10.3691			8.5829	13.2832	3.3296	20.8293
10.5741	10.7631			7.5314	14.0628		
10.3213	10.9652			6.4754	15.0422		
9.7359	11.4273			5.4520	16.7978		
9.4573	11.8174			4.1225	19.1531		
9.2159	11.8894			2.6295	23.4147		

Table S8. Experimental binodal weight fraction data for the systems composed of PEG 600 (1) + NaPA 8000 (2) + water + 2.5 wt% of [Ch]X, X = Cl and [Ac], determined at 300.15K.

[Ch]Cl				[Ch][Ac]			
**100 *w1***	**100 *w2***	**100 *w1***	**100 *w2***	**100 *w1***	**100 *w2***	**100 *w1***	**100 *w2***
29.6776	2.1156	13.6448	7.0901	24.3756	2.9152	10.0362	9.8804
26.0777	2.4486	13.5190	7.2218	23.3797	3.2038	9.3952	10.1686
23.9821	2.8264	13.3243	7.3614	22.7240	3.2194	9.3624	10.4365
21.7580	3.1643	13.1955	7.4897	21.8338	3.6184	9.0611	11.4447
21.6817	3.2976	12.7675	7.7748	20.8342	3.6407	8.0281	12.2205
21.0214	3.4262	12.4112	8.1036	19.7260	4.1170	7.0118	13.9448
19.7696	3.8520	11.9521	8.3230	19.5468	4.2379	5.6270	15.0265
19.2867	4.1178	11.5887	8.5021	18.1224	4.6052	4.6331	18.3442
18.9950	4.1551	11.3073	8.8798	17.5007	5.0399		
18.3678	4.3825	10.7977	9.1568	16.7868	5.1870		
18.1599	4.5712	10.2048	9.4998	16.2257	5.4972		
18.0802	4.5834	9.7665	9.9999	15.4808	5.8008		
17.5848	4.7993	9.5280	10.3049	15.4076	6.0075		
17.4243	4.8166	8.0102	11.6228	14.5772	6.2251		
17.1615	4.9825	7.5713	11.9171	14.3455	6.5046		
16.8446	5.1393	7.2065	12.2980	13.7975	6.6872		
16.7013	5.3058	6.7393	12.6590	13.6026	6.9376		
16.6000	5.3555	6.3636	13.2113	13.0951	7.4091		
16.2403	5.4926	5.9752	13.8277	12.7339	7.4263		
15.7187	5.7442	5.3474	14.3589	12.4609	7.6928		
15.2978	5.9354	4.7927	14.9810	11.9406	8.0407		
15.1719	6.1026	4.4077	15.9317	11.6639	8.3294		
14.9314	6.2885	3.8118	16.9360	11.5966	8.4569		
14.6389	6.4750	3.2162	18.6362	11.2212	8.7950		
14.3080	6.6787	2.4686	20.7526	10.9275	8.9281		
14.0932	6.8984			10.6003	9.3934		

Table S9. Experimental binodal weight fraction data for the systems composed of PEG 600 (1) + NaPA 8000 (2) + water + 2.5 wt% of [Ch]X, X = [DHP], [Bit] and [DHcit], determined at 300.15K.

[Ch][DHP]		[Ch][Bit]		[Ch][DHcit]			
100 *w1*	100 *w2*	100 *w1*	100 *w2*	100 *w1*	100 *w2*	100 *w1*	100 *w2*
48.4052	0.6514	38.0560	2.6711	38.1256	2.2127	16.9202	7.1553
39.1697	1.3816	31.4109	3.1864	33.4958	2.6858	16.3463	7.3697
32.4314	1.7746	26.8374	3.8153	30.6888	2.9078	16.3142	7.4397
29.0554	2.1187	26.5532	4.0584	28.7751	3.2251	15.9326	7.6402
24.4036	3.2898	23.9127	4.3209	27.2878	3.5105	15.6017	7.8093
22.2024	3.9447	23.7192	4.4333	25.6090	3.7999	15.5912	7.8427
21.1886	4.1691	22.2060	4.8198	24.8546	3.9990	15.5405	8.0414
19.8640	4.5427	21.6017	5.1743	24.0053	4.2628	14.6877	8.4119
19.7366	4.7663	21.2426	5.2137	22.4668	4.7181	14.4452	8.7066
19.2505	4.8147	20.2243	5.6645	21.6957	4.8771	14.0338	8.8727
19.0756	4.9146	18.8472	5.9919	21.5741	5.0818	13.9237	8.9325
18.7354	5.0977	18.3591	6.3342	21.0519	5.1199	13.6906	9.0280
18.6701	5.2802	17.4041	6.7259	20.7797	5.2891	13.4776	9.2883
18.4214	5.2825	16.3409	7.0843	20.3360	5.3060	13.0519	9.6308
17.6771	5.6816	16.2930	7.5520	20.1393	5.3853	12.6131	9.9355
17.1730	5.9750	15.3690	8.0543	19.8464	5.5274	12.3253	10.0561
16.9045	6.2674	14.6267	8.8768	19.7785	5.6375	12.1291	10.3837
16.0503	6.5848	10.6126	11.5955	19.7209	5.6752	11.3310	10.7489
15.4969	6.9046	9.6025	12.6109	19.1566	5.8076	10.7664	11.2389
14.7707	7.4838	8.3853	13.6789	19.0180	5.9136	9.9905	11.9503
14.0350	7.7969	7.2142	14.7947	18.9047	6.1008	8.7796	12.6744
13.9763	8.1935	5.4628	15.7811	18.5814	6.1067	8.2214	13.6262
13.0778	8.7481	4.7131	19.4162	18.4113	6.2271	7.5739	14.4080
12.4113	9.3075			18.3170	6.2912	6.4155	15.3259
10.3199	10.9585			18.1290	6.3572	5.2809	16.9510
9.7430	11.6136			17.9483	6.5183	3.9536	18.8896
8.1999	12.6479			17.6931	6.6947		
7.3703	13.4506			17.4005	6.8032		
6.6204	14.4006			17.3349	6.8522		
5.7693	15.4119			17.0689	6.9978		

Table S10. Experimental binodal weight fraction data for the systems composed of PEG 600 (1) + NaPA 8000 (2) + water + 5 wt% of [Ch]X, X = Cl and [Ac], determined at 300.15K.

[Ch]Cl				[Ch][Ac]			
**100 *w1***	**100 *w2***	**100 *w1***	**100 *w2***	**100 *w1***	**100 *w2***	**100 *w1***	**100 *w2***
25.1491	2.3364	5.6207	13.6698	21.4017	3.7864	7.7252	11.8046
21.3523	3.1556	5.2406	14.5508	19.2553	3.9999	6.6225	13.3984
20.5117	3.5010	4.6566	15.1248	18.9732	4.0378	5.5533	15.0832
19.5203	3.7108	4.0526	16.1773	17.9705	4.3047	4.3823	18.3445
18.7926	4.0016	3.4361	17.9787	17.7180	4.3110		
18.1432	4.2878	2.6852	19.4121	17.5451	4.4039		
17.2884	4.6484	1.9918	20.3835	16.7975	4.6627		
16.8672	4.7913			16.6444	4.7474		
16.1764	5.1497			15.8893	5.0285		
15.6390	5.4029			15.1910	5.3814		
14.8483	5.8827			15.1512	5.4554		
14.3791	6.1344			14.3308	5.8885		
13.8735	6.4168			14.3043	5.8977		
13.3383	6.7710			13.7125	6.3169		
11.2692	8.4250			13.1869	6.5189		
10.8306	8.6766			12.8489	6.6510		
10.5197	8.9421			12.7660	7.0384		
10.1831	9.1599			12.1765	7.1943		
9.9022	9.3963			11.7017	7.4732		
9.7855	9.5786			11.5019	7.7788		
9.5854	9.9409			11.3975	7.9332		
9.2636	10.2435			10.9419	8.2892		
8.8711	10.5180			10.3918	8.6923		
8.4300	10.8399			10.3231	8.7050		
7.9695	11.2180			9.7496	9.0447		
7.5918	11.4997			9.2713	9.6181		
7.3320	12.1887			9.2632	9.7591		
6.4983	13.1027			8.7851	10.9494		

Table S11. Experimental binodal weight fraction data for the systems composed of PEG 600 (1) + NaPA 8000 (2) + water + 5 wt% of [Ch]X, X = [DHP], [DHcit] and [Bit], determined at 300.15K.

[Ch][DHP]		[Ch][DHcit]				[Ch][Bit]			
100 *w1*	100 *w2*	100 *w1*	100 *w2*	100 *w1*	100 *w2*	100 *w1*	100 *w2*	100 *w1*	100 *w2*
24.4297	3.1373	57.6120	0.7478	17.0602	7.9706	62.2136	1.7814	14.6365	9.8653
21.0999	4.5286	34.3054	2.7416	16.8770	8.0700	52.5733	2.3021	14.0050	10.2305
18.8096	5.3104	31.3698	3.2824	16.6071	8.2083	44.6771	3.2771	12.6883	11.1395
16.8888	6.1427	30.1640	3.4983	16.2660	8.4998	37.3126	3.8869	11.2239	11.7630
15.0411	6.9715	28.0897	3.9969	16.0804	8.5396	32.3674	4.2323	10.6607	12.5969
11.7951	9.3382	26.4449	4.3812	15.6927	8.6625	29.9647	4.4224	9.2180	13.6766
11.5536	9.5282	25.9101	4.4693	15.2476	8.9554	28.6764	4.6788	8.0577	15.5889
9.8168	10.9834	24.8802	4.7702	14.9280	9.3237	27.0796	4.9042	4.8035	19.3919
8.3572	12.1637	24.3640	4.8088	14.3502	9.4292	25.4448	5.4349		
8.1333	12.3493	23.7758	5.0868	14.2403	9.7241	24.3991	5.5574		
6.2687	14.3867	22.9845	5.3585	14.0816	9.7553	23.2431	5.9013		
4.2163	16.5466	22.8497	5.4682	13.7589	9.9232	23.1088	6.1244		
3.4545	17.8155	22.2941	5.7055	12.9219	10.2577	21.9647	6.3742		
		22.1434	5.7341	12.6329	10.4988	21.9000	6.4050		
		21.0172	6.0988	11.8999	10.9395	21.0747	6.7205		
		20.5719	6.3000	11.5913	11.2237	20.8167	6.7377		
		20.3958	6.4215	10.6640	11.8196	20.4978	6.9869		
		20.1778	6.4756	10.4120	12.1759	19.7306	7.1758		
		19.7313	6.6378	9.7228	12.6220	19.4539	7.3749		
		19.1847	6.9432	9.2715	13.0348	18.7232	7.6140		
		19.1301	7.0480	8.5963	13.4540	18.6921	7.7185		
		18.7089	7.1195	7.5510	14.1189	18.0215	7.9331		
		18.5329	7.2034	7.1311	14.5535	17.9061	7.9695		
		18.2212	7.2600	6.3375	15.7635	17.1094	8.3706		
		18.0815	7.4733	5.9758	16.6236	16.5921	8.6832		
		17.9678	7.5607	4.1325	18.8669	16.4422	8.8648		
		17.6495	7.6487			15.5315	9.2420		
		17.2403	7.8416			14.9597	9.4808		

Table S12. Experimental binodal weight fraction data for the systems composed of PEG 600 (1) + NaPA 8000 (2) + water + 10 wt% of [Ch]X, X = Cl and [Ac], determined at 300.15K.

[Ch]Cl				[Ch][Ac]			
100 *w1*	100 *w2*	100 *w1*	100 *w2*	100 *w1*	100 *w2*	100 *w1*	100 *w2*
33.8098	1.2398	8.1705	10.4166	27.8541	2.1921	11.1646	7.2578
29.0288	1.6864	8.0353	10.5816	23.7725	2.6356	11.1336	7.4373
24.1185	2.3956	7.8069	10.7905	21.0038	3.2577	10.8658	7.6617
22.4232	2.6884	7.6421	11.1562	19.0572	3.7062	10.6459	7.6953
20.9179	3.0110	7.3157	11.4379	17.4765	4.2566	10.6348	8.0266
19.8901	3.2123	7.0162	11.6977	16.0626	4.6955	10.1059	8.3503
19.1240	3.4469	6.7447	11.9426	15.1802	4.9763	9.7746	8.8310
18.5193	3.6591	6.5595	12.4101	14.8770	5.2821	9.3868	9.2572
17.8678	3.9131	6.2415	12.7316	14.0971	5.4846	8.7534	10.0790
17.3037	4.1315	5.9018	13.0505	14.0638	5.6902	7.9924	10.9372
16.6415	4.3996	5.5615	13.6539	13.2902	6.0645	7.1675	11.4964
16.1184	4.6542	5.0932	14.0409	12.8598	6.1815	6.4608	12.3581
15.6775	4.8248	4.8981	14.6600	12.7232	6.2690	5.6129	13.0196
15.0979	5.1190	4.3832	15.4369	12.5508	6.4575	5.3527	13.9997
14.7138	5.2535	3.8651	16.2147	12.3397	6.4896	4.4551	14.8685
14.0937	5.6117	3.4888	17.0616	12.2866	6.6947	3.9074	17.8842
13.5232	5.8942	2.9925	18.0287	11.7350	6.9114	2.1795	19.3297
12.9339	6.2679	2.5739	19.0805	11.5904	6.9924		
8.4247	10.2259			11.5075	7.1677		

Table S13. Experimental binodal weight fraction data for the systems composed of PEG 600 (1) + NaPA 8000 (2) + water + 10 wt% of [Ch]X, X = [DHP], [DHcit] and [Bit], determined at 300.15K.

[Ch][DHP]		[Ch][DHcit]				[Ch][Bit]	
100 *w1*	100 *w2*	100 *w1*	100 *w2*	100 *w1*	100 *w2*	100 *w1*	100 *w2*
24.2622	3.7772	57.7008	0.7470	18.2422	9.1211	53.9838	2.0020
22.5645	4.3317	43.6067	2.2085	17.5090	9.5403	29.0091	5.5580
19.2803	5.1759	41.8212	2.5107	16.6847	9.6117	25.4684	6.3242
18.1361	5.7163	37.1924	3.0831	16.2740	10.0302	23.9590	6.7033
16.2240	6.6013	34.4426	3.6504	14.4220	10.9499	22.6321	7.1083
14.4133	7.4698	34.0532	3.7162	13.1445	11.8696	21.0374	7.5796
13.0852	8.0834	32.4130	4.0329	12.4994	12.1696	19.6465	8.1072
12.8711	8.2794	32.2996	4.1263	11.4569	12.8258	18.6153	8.5936
11.5513	9.0994	31.5820	4.3774	8.3868	14.6167	18.2847	8.6814
10.1235	10.0553	29.3701	4.5725	7.9932	15.4458	16.5173	9.4150
9.9401	10.0842	29.2441	4.8510	7.3632	16.3044	16.5084	9.4426
8.8776	10.9433	28.0329	5.1974	6.1353	17.5624	15.3194	9.9462
8.0100	11.5083	27.3236	5.2199			15.2765	10.1471
7.6770	11.8974	26.4838	5.6045			14.3184	10.6612
7.1653	12.2866	25.5004	5.9582			13.9177	10.6845
6.6981	12.7102	24.9494	5.9719			13.1460	11.2538
6.3612	12.8534	24.8973	6.1904			12.6974	11.2884
5.8344	13.4980	24.0957	6.5038			11.9484	11.7203
5.7456	13.6158	23.0773	6.9327			11.9304	11.8820
4.6166	14.8434	22.6083	7.1602			10.5957	12.5898
4.1600	15.2644	21.9295	7.4383			10.4950	12.6188
3.7230	16.1346	21.0814	7.4995			9.8048	13.0426
2.5807	18.4114	21.0100	7.7438			9.1006	13.7079
		20.5494	7.9938			8.3796	14.3174
		20.0131	8.2012			7.4384	15.1124
		19.4158	8.2610			6.5307	16.0870
		19.3408	8.4990			5.4420	17.2158
		18.7788	8.8020			4.4598	18.6537

Table S14. Experimental binodal weight fraction data for the systems composed of PEG 600 (1) + NaPA 8000 (2) + water, determined at 300.15K.

0% electrolyte			
100 *w1*	100 *w2*	100 *w1*	100 *w2*
28.1862	2.3155	**11.5343**	10.3369
26.3684	2.4134	11.1387	10.5996
24.4414	3.1864	11.1088	10.8985
21.7966	3.8956	10.6981	10.9633
20.0505	4.7358	10.4649	11.4550
19.0319	4.9585	10.2736	11.5971
17.9428	5.5830	9.7014	12.3906
16.8543	6.1602	8.5098	13.0199
15.9753	6.8495	8.0010	13.4899
14.9248	7.5844	7.6666	14.0553
14.1999	8.1486	7.1280	14.6505
14.0782	8.1741	6.4816	15.2321
13.8352	8.4629	6.0404	15.8321
13.4004	8.8689	5.4144	16.6881
12.9887	9.0204	4.6406	17.5635
12.9746	9.3627	3.7826	18.3779
12.3537	9.7816	3.2772	19.8021
12.0381	9.8544	2.3425	22.2530
11.7856	10.2621	1.2223	26.8263

Table S15. Experimental binodal weight fraction data for the systems composed of PEG 600 (1) + NaPA 8000 (2) + X wt% NaCl + water, determined at 300.15K.

1% NaCl		2.5% NaCl		5% NaCl		10% NaCl	
100 ***w1***	100 ***w2***	100 ***w1***	100 ***w2***	100 ***w1***	100 ***w2***	100 ***w1***	100 ***w2***
17.2277	1.5059	10.4369	2.4620	9.7524	1.1381	6.9431	1.8493
16.8456	1.6611	10.1728	2.6386	6.8700	3.0212	6.6506	2.1035
16.4354	1.8353	10.1459	2.7806	6.7609	3.2235	6.3935	2.4682
16.3977	2.0065	9.8901	2.9225	6.6485	3.3607	6.3454	2.6134
16.1190	2.1954	9.8368	3.0401	6.6475	3.5243	6.2873	2.8898
15.6502	2.4266	9.6628	3.0704	6.6038	3.6724	6.2315	3.2978
15.0973	2.6427	9.6325	3.2331	6.5173	3.8395	6.1299	3.5028
14.8733	2.8979	9.4061	3.3817	6.4152	4.0290	6.1156	3.6748
14.7071	3.0860	9.2592	3.4786	6.3559	4.0727	6.1001	3.8034
14.2942	3.2562	9.1993	3.5823	6.3523	4.2108	6.0451	3.9976
14.2942	3.5151	9.1268	3.6988	6.3005	4.3604	6.0377	4.1979
14.1486	3.5854	9.1150	3.7033	6.2874	4.5180	6.0259	4.4846
13.6075	3.8082	9.0902	3.8325	6.2630	4.5827	6.0238	4.7984
13.4239	4.0738	9.0513	4.0224	6.2612	4.7656	5.9965	4.9936
13.1657	4.5253	8.7709	4.2629	6.2071	4.9325	5.9468	5.3306
12.1490	5.2624	8.7383	4.4183	6.1371	5.0532	5.9416	5.6081
11.9073	5.3613	8.6878	4.6065	6.1083	5.1791	5.9248	5.8107
10.8083	6.3419	8.5615	4.7943	6.0770	5.3271	5.8545	6.2596
9.6893	7.5211	8.3995	4.9919	6.0606	5.4523	5.8133	6.6605
9.6522	8.0889	8.2832	5.2292	6.0320	5.5932	5.7491	7.1507
8.5663	8.9005	8.1202	5.4726	5.9968	5.7574		
8.4481	8.9575	7.8924	5.7462	5.9918	5.9227		
7.7732	10.0301	7.7878	6.1399	5.9521	6.2681		
7.4299	10.1247	7.6021	6.5350	5.9379	6.4509		
6.6785	11.0775	7.4205	7.0039				
6.6268	11.2249	7.0262	7.3023				
5.8685	12.1191	6.8127	7.6839				
5.8053	13.2133	6.6898	8.4605				
		6.3199	9.1702				

Table S16. Experimental binodal weight fraction data for the systems composed of PEG 600 (1) + NaPA 8000 (2) + X wt% NaOAc + water, determined at 300.15K.

1% NaOAc		2.5% NaOAc		5% NaOAc		10% NaOAc	
100 $w1$	100 $w2$	100 $w1$	100 $w2$	100 $w1$	100 $w2$	100 $w1$	100 $w2$
19.3517	1.4499	11.5672	2.0871	7.5842	1.7595	4.0474	2.5347
17.8943	2.1739	11.0203	2.6575	1.9108	20.3352	3.7360	2.8020
15.9907	3.4263	10.7207	2.8393	3.3660	15.5819	3.7144	3.3493
15.7231	3.4781	10.6557	2.9011	3.9943	12.3599	3.6471	4.1339
15.3646	3.7041	10.5358	3.0224	4.4034	10.2249	3.6225	5.2654
14.7547	3.9547	10.4570	3.2040	4.8282	8.9778	3.5464	7.4493
14.6236	4.1613	10.2263	3.4139	5.0807	7.9204	3.3746	8.7767
14.4616	4.3676	9.9893	3.6382	5.2460	6.9657	3.3624	12.7964
13.9382	4.8350	9.8233	3.9412	5.4476	6.3581	2.4683	19.1114
13.6525	5.1459	9.4839	4.1035	5.5664	5.8610		
13.1074	5.3799	9.3613	4.2687	5.7384	5.4368		
12.6583	5.6362	9.2646	4.4158	5.9018	5.0673		
12.5262	5.8883	8.9903	4.6895	5.8969	4.6454		
12.4174	6.1020	8.7750	4.8478	5.9499	4.3674		
12.2418	6.3194	8.6162	5.1112	6.0730	4.1337		
12.0004	6.5790	8.5871	5.3586	6.2211	3.9123		
11.2504	6.9831	8.5174	5.4131	6.1894	3.6714		
11.2040	7.0145	8.4119	5.7421	6.5471	3.4646		
10.9142	7.3311	8.0727	5.9663	6.7512	3.2108		
10.6580	7.6418	7.9605	6.3732	6.7478	2.9059		
10.2744	8.0149	7.8086	6.5464	6.7741	2.7172		
9.8161	8.4479	7.6223	6.8901				
9.3218	8.8936	7.3922	7.1490				
9.1732	8.9992	7.3542	7.4286				
8.9360	9.4231	7.0811	8.0175				
8.4234	10.0569	6.6180	8.4318				
7.6839	10.5676	6.5579	8.5931				
7.6387	10.6748	6.2344	9.4226				
7.0424	11.4436	5.9438	9.4322				
6.3855	12.2480	5.7305	10.4384				
5.5832	13.2887	5.1132	11.6585				
4.8133	15.3705	4.4004	13.2560				
3.6412	17.9291	3.6060	16.2482				
2.0918	19.8026	2.2504	20.7383				

Table S17. Correlation parameters to describe the binodal curve, where *A*, *B* and *C* are constants obtained by the regression of the experimental binodal data through the application of Eq. (1) (and respective standard deviations, σ, and correlation coefficients, R^2) for the system composed of PEG 600 + NaPA 8000.

0 wt % of [Ch]X	
R^2	0.9977
$A \pm \sigma$	54.17 ± 2.03
$B \pm \sigma$	-0.45 ± 0.02
$10^5 C \pm \sigma$	9.65 ± 1.28

Table S18. Correlation parameters to describe the binodal curves, where *A*, *B* and *C* are constants obtained by the regression of the experimental binodal data through the application of Eq. (1) (and respective standard deviations, σ, and correlation coefficients, R^2) for the systems composed of PEG 600 + NaPA 8000 + [Ch]Cl.

	1 wt % of [Ch]Cl	2.5 wt % of [Ch]Cl	5 wt % of [Ch]Cl	10 wt % of [Ch]Cl
R^2	0.9954	0.9938	0.9990	0.9971
$A \pm \sigma$	44.31 ± 1.35	62.76 ± 1.79	56.58 ± 0.87	68.84 ± 1.48
$B \pm \sigma$	-0.37 ± 0.01	-0.57 ± 0.01	-0.54 ± 0.01	-0.67 ± 0.01
$10^5C \pm \sigma$	18.14 ± 1.31	8.24 ± 1.30	9.58 ± 0.60	2.11 ± 1.16

Table S19. Correlation parameters to describe the binodal curves, where *A*, *B* and *C* are constants obtained by the regression of the experimental binodal data through the application of Eq. (1) (and respective standard deviations, σ, and correlation coefficients, R^2) for the systems composed of PEG 600 + NaPA 8000 + [Ch][Ac].

	1 wt % of [Ch][Ac]	2.5 wt % of [Ch][Ac]	5 wt % of [Ch][Ac]	10 wt % of [Ch][Ac]
R^2	0.9972	0.9977	0.9920	0.9941
$A \pm \sigma$	62.07 ± 2.36	69.26 ± 1.41	71.75 ± 3.40	75.18 ± 2.33
$B \pm \sigma$	-0.53 ± 0.02	-0.62 ± 0.01	-0.66 ± 0.02	-0.70 ± 0.01
$10^5C \pm \sigma$	7.90 ± 0.99	0.83 ± 0.88	-1.42 ± 1.61	1.31 ± 1.42

Table S20. Correlation parameters to describe the binodal curves, where *A*, *B* and *C* are constants obtained by the regression of the experimental binodal data through the application of Eq. (1) (and respective standard deviations, σ, and correlation coefficients, R^2) for the systems composed of PEG 600 + NaPA 8000 + [Ch][DHP].

	1 wt % of [Ch][DHP]	2.5 wt % of [Ch][DHP]	5 wt % of [Ch][DHP]	10 wt % of [Ch][DHP]
R^2	0.9986	0.9920	0.9988	0.9994
$A \pm \sigma$	55.87 ± 1.65	78.45 ± 1.91	59.03 ± 2.08	78.88 ± 1.94
$B \pm \sigma$	-0.47 ± 0.01	-0.63 ± 0.01	-0.49 ± 0.02	-0.60 ± 0.01
$10^5C \pm \sigma$	10.14 ± 1.12	0.00 ± 2.51	13.68 ± 1.22	1.56 ± 0.80

Table S21. Correlation parameters to describe the binodal curves, where *A*, *B* and *C* are constants obtained by the regression of the experimental binodal data through the application of Eq. (1) (and respective standard deviations, σ, and correlation coefficients, R^2) for the systems composed of PEG 600 + NaPA 8000 + [Ch][DHcit].

	1 wt % of [Ch][DHcit]	2.5 wt% of [Ch][DHcit]	5 wt % of [Ch][DHcit]	10 wt % of [Ch][DHcit]
R^2	0.9985	0.9918	0.9980	0.9984
$A \pm \sigma$	61.65 ± 2.49	92.46 ± 2.63	95.45 ± 0.97	90.76 ± 0.96
$B \pm \sigma$	-0.51 ± 0.02	-0.64 ± 0.01	-0.60 ± 0.01	-0.51 ± 0.01
$10^5C \pm \sigma$	6.48 ± 1.35	2.33 ± 1.34	6.54 ± 0.68	11.69 ± 0.79

Table S22. Correlation parameters to describe the binodal curves, where *A*, *B* and *C* are constants obtained by the regression of the experimental binodal data through the application of Eq. (1) (and respective standard deviations, σ, and correlation coefficients, R^2) for the systems composed of PEG 600 + NaPA 8000 + [Ch][Bit].

	1 wt % of [Ch][Bit]	2.5 wt % of [Ch][Bit]	5 wt % of [Ch][Bit]	10 wt % of [Ch][Bit]
R^2	0.9782	0.9865	0.9917	0.9996
$A \pm \sigma$	105.68 ± 8.30	122.12 ± 9.17	196.48 ± 6.83	138.99 ± 1.29
$B \pm \sigma$	-0.72 ± 0.04	-0.76 ± 0.04	-0.87 ± 0.02	-0.67 ± 0.00
$10^5C \pm \sigma$	-1.61 ± 2.78	-2.64 ± 2.69	-5.40 ± 2.14	9.74 ± 0.44

Table S23. Number of moles of $[Ch]^+$ at each phase, the volume of the phase, and *Kp* values of $[Ch]^+$.

	[Ch]Cl		[Ch][Ac]		[Ch][DHP]		[Ch][DHcit]		[Ch][Bit]	
Phase	top	bot	top	bot	top	bot	top	bot	top	bot
**n $[Ch]^+$ (mol)**	7.33E-03	3.71E-05	6.72E-03	3.65E-05	6.18E-03	7.93E-05	7.42E-03	5.30E-04	7.85E-03	4.56E-04
**Volume(mL)**	6.5	2	7	2	5.9	2.9	7	1.7	7	1.9
Kp	60.89		52.54		38.28		3.40		4.68	

Table S24. Concentration of chloride anion at each corresponding phase (determined using a chloride-selective electrode), the absolute values of the integral of the ^1H RNM peaks of the anions, and *Kp* values of the anion for each electrolyte in the ABS. X^- is the chloride, acetate, bitartrate and dihydrogencitrate anions of the added electrolytes.

	NaCl		[Ch]Cl		Na[Ac]		[Ch][Ac]		[Ch][DHcit]		[Ch][Bit]	
Phase	top	bot	top	bot	top	bot	top	bot	top	bot	top	bot
**Cl^- conc. (mmol/L)**	2175	1275	900	300	-	-	-	-	-	-	-	-
**Integrals of RMN peaks**	-	-	-	-	1.1E+08	5.77E+07	4.82E+07	1.90E+07	1.11E+08	5.25E+07	1.21E+08	6.56E+07
Kp X^-	1.71		3		1.90		2.53		2.21		1.86	

Table S25. Concentration of sodium cation at each corresponding phase (determined using a sodium-selective electrode), *Kp* and log Kp values of sodium cation in studied ABSs.

	[Ch]Cl		[Ch][Ac]		[Ch][DHcit]		[Ch][Bit]		NaCl		Na[Ac]		Without electrolyte	
Phase	top	bot	top	bot	top	bot	top	bot	top	bot	top	Bot	top	bot
**Na^+ conc. (mmol/L)**	550	4550	575	4950	1250	3325	1225	4200	4500	10700	2925	8750	250	7250
**Kp**	0.12		0.12		0.38		0.29		0.42		0.33		0.03	
Log Kp	-0.92		-0.93		-0.42		-0.54		-0.38		-0.48		-1.46	
